# Integrated Molecular Screening and Process Optimization To Identify Ionic Liquids for Energy-Efficient Refrigerant Separation

**DOI:** 10.1021/acssuschemeng.5c05520

**Published:** 2025-09-10

**Authors:** Ashfaq Iftakher, Mohammed Sadaf Monjur, Ahaduzzaman Nahid, Michael Dukissis, M. M. Faruque Hasan

**Affiliations:** † Artie McFerrin Department of Chemical Engineering, 14736 Texas A&M University, College Station, Texas 77843-3122, United States; ‡ Texas A&M Energy Institute, 14736 Texas A&M University, College Station, Texas 77843, United States

**Keywords:** hydrofluorocarbons (HFC) reclamation, solvent screening, ionic liquid, extractive distillation, computer-aided molecular and process design

## Abstract

Hydrofluorocarbon (HFC)-based mixed refrigerants are widely used in commercial cooling and manufacturing processes. HFCs have a high global warming potential (GWP), which makes their reclamation particularly important. End-of-life recovery of HFCs that form azeotropes requires advanced separation technologies. Solvent-based extractive distillation can break azeotropes and recover high-purity constituents, but solvent selection critically affects the separation performance. In this work, we present a computer-aided molecular and process design (CAMPD) framework that integrates molecular simulation and solubility-based screening with rigorous process optimization to identify promising ionic liquid solvents for HFC separation. This approach addresses the complex multiscale interplay between solvent choice and the operating conditions of the extractive distillation process, offering a holistic solution to the HFC separation challenge. We apply the framework for the separation of R-410A, a 50/50 wt % blend of HFC-32 and HFC-125. We screen 341,687 ionic liquids and salts, the largest set of solvent candidates considered for this application. We identify 285 new ionic liquids that outperform the existing solvents for the R-410A separation. Many show potential to significantly reduce the process energy consumption of HFC separation. We also analyze the molecular features of the top-performing ionic liquids to gain insights and uncover design principles for their use as effective mass separating agents.

## Graphical abstract

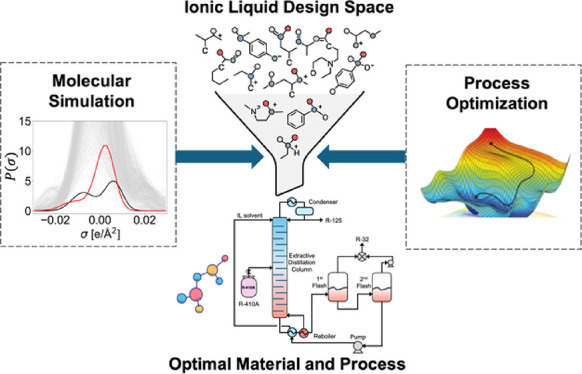

## 1 Introduction

Refrigerants are essential to everyday life with applications in household and commercial cooling systems, food safety, and transportation. The combined annual refrigerant market value exceeds 14 billion USD, with more than 800 million kilograms of refrigerants in use for cooling applications.^1^ These are expected to increase further with the rise in global energy demand and industrialization.^2^ Chlorofluorocarbons (CFCs) and hydrochlorofluorocarbons (HCFCs) were early-generation refrigerants, but they caused severe ozone depletion, prompting the 1987 Montreal Protocol to mandate their phase-out.^3^ This led to the adoption of hydrofluorocarbons (HFCs) as the third generation of refrigerants. Despite having zero ozone depletion potential (ODP), HFCs are potent greenhouse gases with alarmingly high global warming potentials (GWP).^4,5^ Commonly used refrigerants often contain HFC-125 and HFC-32, which have GWP of 3500 and 675, respectively (see Figure 1a). Realizing the detrimental effect of HFC release into the atmosphere, major legislations, such as the Kigali Amendment to the Montreal Protocol^6^ and the American Innovation and Manufacturing (AIM) Act,^7^ were enacted calling for the phase out (by at least 85%) of HFCs by 2036 and a transition to the next generation of refrigerants such as hydrofluoroolefins (HFOs) with zero ODP and reduced GWP. This transition requires massive reclamation, recovery, and recycling of HFCs. R-410A is the most widely used HFC-based mixture of HFC-32 and HFC-125. HFC-32 and HFC-125 are also known as R-32 and R-125, respectively. Reclaimed R-32 can be blended with low-GWP HFOs to create new refrigerants (such as R-447A, R-452B, and R-454),^8^ while R-125 can serve as a feedstock for producing valued chemicals, such as fluoropolymers.^9^ Overall, this would ultimately reduce the need to produce virgin HFC components.^10,11^ Only less than 3% of refrigerants that enter the market today are recovered at their end-of-life.^12^ Therefore, there is a need for innovative separation processes that can efficiently recover high-purity refrigerant components. Typical impurities such as water and lubricants can be removed from the refrigerant mixtures at end-of-life using fractional distillation or flash separations, but it is difficult to separate pure R-32 and R-125 from their mixtures using conventional distillation due to azeotrope formation.

**Figure 1:**
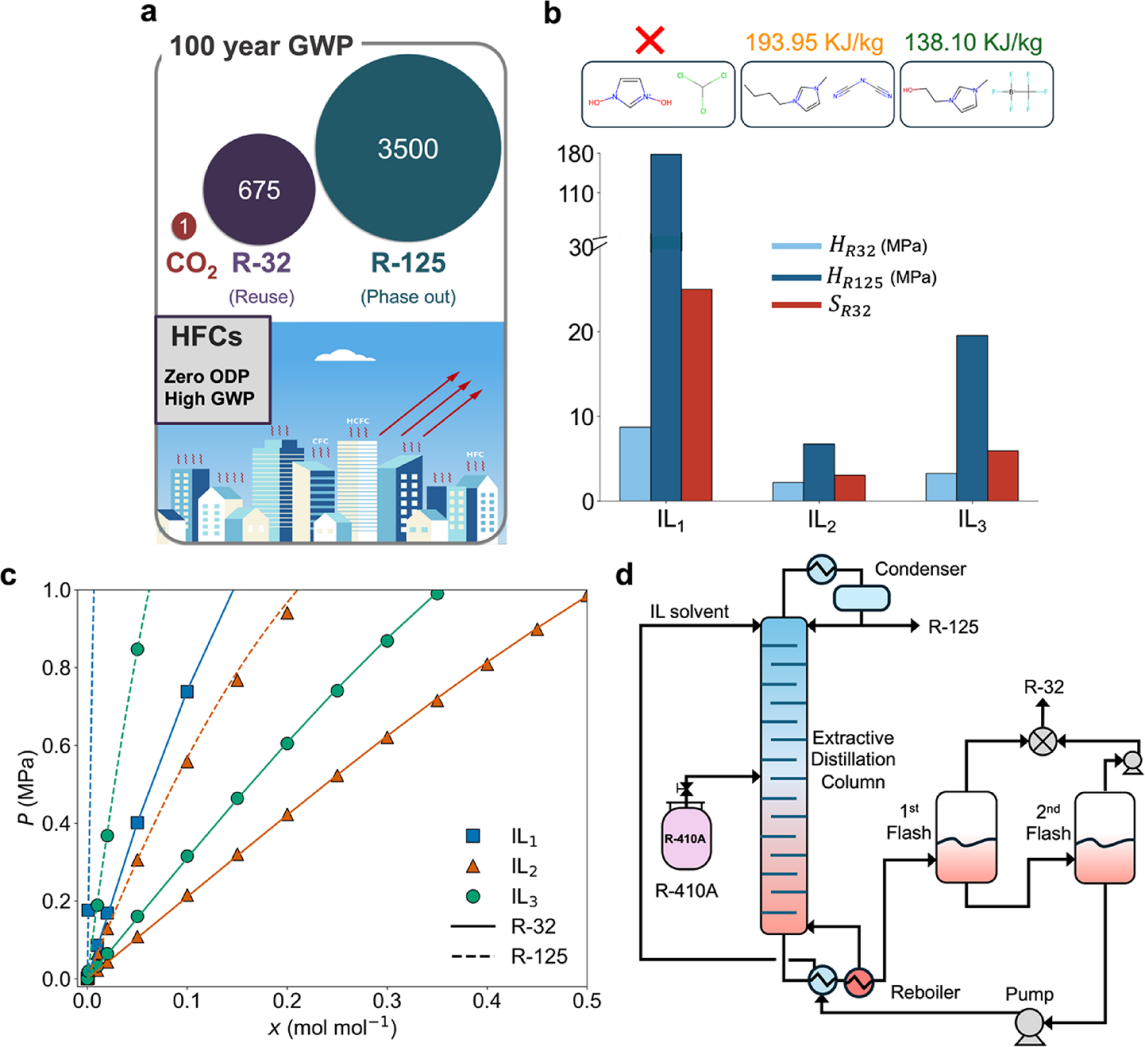
HFC separation using ionic liquid-based extractive distillation and the need for integrated molecular screening and process optimization. (a) R-32 and R-125 have orders of magnitude more GWP than CO_2_ and (b) comparison of three indicative ILs in terms of their solubility measures and separation performance. IL_1_ is 1,3-dihydroxylimidazolium CHCl_3_ and is unable to attain the 99.5 wt % purity requirement for recovered R-32 and R-125. IL_2_ is 1-butyl-3-methylimidazolium dicyanamide and requires an equivalent work of 193.95 kJ/kg to separate R-410A with a minimum required purity. IL_3_ is 1-(2-hydroxyethyl)-3-methylimidazolium trifluoromethyltrifluoroborate and requires the least amount of work among the three indicative ILs with 138.10 kJ/kg to separate 1 kg of R-410 with minimum required purity. Ideal R-32 selectivity is defined as $S_{R32}=\frac{\gamma_{R125}^{\infty }}{\gamma_{R32}^{\infty }}$ , where γ_*R*125_^∞^ and γ_*R*32_^∞^ are infinite dilution activity coefficients of R-32 and R-125 in IL at 298.15 K. (c) COSMO-RS derived equilibrium solubility data at 298.15 K indicates IL_2_ > IL_3_ > IL_1_ in terms of R-32 solubility (absorption is inverse to the slope or Henry’s constant), but IL_1_> IL_3_ > IL_2_ in terms of R-32 selectivity. (d) Extractive distillation process configuration for R-410A separation indicates IL_3_ > IL_2_ > IL_1_ in terms of process energy intensity.

Extractive distillation has emerged as a viable strategy for azeotropic mixture separation.^9,13,14^ In extractive distillation, a heavy-boiling entrainer (solvent) is introduced to selectively absorb one component over the others and break the azeotrope. Recently, ionic liquids (ILs) have gained significant attention as green solvents for extractive distillation. They offer a unique suite of properties that are highly attractive for separation processes. These include negligible vapor pressure (hence, negligible solvent losses and contamination), high thermal and chemical stability, nonflammability, and high chemical tunability. Prior studies have reported excellent solubility of refrigerant gases (including HFCs) in ILs.^12,15^ An IL is made of a cationic core, an anion, and functionalized/nonfunctionalized alkyl side chains. By selecting appropriate cations and anions, one can achieve favorable changes in properties such as volatility, viscosity, and gas solvation capacity. This tunability has earned ILs the nickname “designer solvents”.^16^ Unlike adsorption-based separation that uses microporous materials^17^ for periodic adsorption and desorption of HFCs, ILs allow extractive distillation to operate at a steady state continuous mode. However, identifying the optimal IL is a challenging task. More than 2500 ILs have been synthesized to date,^18^ and there are millions of potential ILs.^19^ In the context of refrigerant separation, only a handful of ILs have been studied.^5^ Interestingly, most of these have imidazolium-based cations and one of the three anions, namely, tetrafluoroborate, hexafluorophosphate, and bis­(trifluormethylsulfonyl)­imide.^12^

The large molecular design space of ILs poses several challenges. Experimentally synthesizing and testing a large number of ILs is impractical. Traditional IL selection uses heuristics and solubility measures, which may not translate into the best performance, in terms of process energy intensity and separation cost. Even if a promising IL is identified, designing the optimal process for that IL and finding optimal operating conditions of the extractive distillation process is complex.^13,20,21^ The multiscale interplay between molecular properties and process performance needs to be considered.^22–24^ In the context of R-410A separation, notable works include gas solubility or selectivity-based screening^25^ and simulation of extractive distillation with a few ILs.^9^ To that end, computer-aided molecular and process design (CAMPD) has recently emerged as a systematic technique for solvent screening that aims to integrate molecular and process scale decisions within an equation-oriented optimization framework.^24,26^ As an indicative example, we recently reported a high-throughput CAMPD strategy to select optimal ILs for R-410A separation.^27^ These efforts provide valuable information, yet an integrated screening methodology to handle the enormous IL design space while accounting for process-level performance is missing. Clearly, there is much room (and need) for screening ILs, especially if we aim to reduce the energy intensity of the HFC separation.

In this work, we develop a multiscale framework that combines molecular simulation and process optimization for screening ILs for the R-410A separation. By integrating aspects of molecular simulation, machine learning, and rigorous process optimization, we effectively navigate a vast molecular design space. Specifically, we consider 683 cations and 505 anions, totaling 341,687 ILs and salts as potential solvent candidates for the extractive distillation of R-410A. We employ COSMO-RS^28,29^ to estimate the infinite dilution activity coefficients for R-410A constituents (R-32 and R-125) in each of these candidates. By adopting systematic solubility and selectivity-based criteria and constraints, we progressively refine the choice of a *reference* IL that allows for rapid screening of the most promising ILs without the need for exhaustive process optimization for all IL candidates. For each screened IL, we optimally design an extractive distillation process with minimum process energy intensity for R-410A separation. Reduced process energy intensity also reduces the cost and associated CO_2_ emissions of the separation process. Our results reveal many new ILs achieving energy-efficient separation, outperforming the current-best IL for R-410A separation. We also analyze the molecular features of the top-performing ILs and uncover design principles at the cation/anion level (in terms of the intermolecular forces) to provide an understanding of the superior performance of these ILs. This large-scale screening of the expansive search space involving hundreds of thousands of ILs is, to our knowledge, the largest molecular-to-process scale screening of ILs conducted for refrigerant separation to date. The major contributions of this work include:

- Development of a multiscale CAMPD framework that integrates molecular simulation, solubility-based screening, and process optimization toward the discovery of new ILs for refrigerant separation,
- Starting from a pool of 341,687 ILs and salts, our solubility-based screening discards 99% of candidates without requiring process optimization, allowing for tractable evaluation of only the 355 most promising ILs. The unprecedented success of our CAMPD approach is highlighted by the fact that 80% of these (i.e., 285 out of 355 ILs) are predicted to perform better than the existing solvents,
- Identification of new ILs with superior process performance with at least 13% reduction in the equivalent work for R-410A separation compared to existing solvents, and
- Analysis of molecular surface charge distribution to reveal ILs with weakly electronegative, chloride-rich anions paired with short-chain alkoxy-substituted cations that maximize R-32 absorption while repelling R-125, thereby offering new design rules for IL solvents.

The remainder of the article is organized as follows. In Section 2, we report the need for an integrated molecular screening and process optimization framework. In Section 3, we provide a detailed description of the developed multiscale CAMPD framework. In Section 4, we discuss the screening and process optimization results, leading to the discovery of new ILs. We also investigate the molecular features of the top performing ILs, followed by concluding remarks in Section 5.

## 2 Need for Integrated Molecular Screening and Process Optimization

The development of IL-based separation technology involves: (i) selection of an IL, (ii) design of an extractive distillation process configuration, and (iii) identification of optimized operating conditions. The selection of an IL solvent dictates the energy intensity, cost, and carbon footprint of the extractive distillation process. Solvent selection based on material-centric metrics (e.g., selectivity) alone is not sufficient and may lead to suboptimal and even infeasible process designs. To illustrate this, consider the indicative IL solvents (IL_1_, IL_2_, and IL_3_) shown in Figure 1b. IL_1_ (1,3-dihydroxylimidazolium CHCl_3_) is a computer-generated IL, IL_2_ (1-butyl-3-methylimidazolium dicyanamide) is a well-known solvent [BMIM]­[DCA] for R-410A separation with measured experimental data, and IL_3_ (1-(2-hydroxyethyl)-3-methylimidazolium trifluoromethyltrifluoroborate) is the most energy-efficient IL for R-410A separation identified in the literature to date.^27^ The equilibrium solubilities of R-32 and R-125 in the three ILs at 298.15 K are shown in Figure 1c. The slope at the origin is proportional to Henry’s constants. IL_3_ absorbs nearly three times more R-32 at dilute conditions than IL_1_. R-125 remains poorly absorbed.

We use the SPICE_ED framework^13^ to compare their process performance as a solvent in an extractive distillation process (Figure 1d) to separate R-32 and R-125 from a 50/50 wt % mixture of R-410A. IL_1_ has an ideal R-32 selectivity of 24.99, which is far more than IL_2_ (3.07) and IL_3_ (5.96). However, it is not able to meet the minimum required purity (99.5 wt % pure R-32 and R-125 to be recovered). This is primarily because IL_1_ exhibits a *H*_*R*32_ value of 8.73 MPa, indicating low R-32 absorption. IL_2_ and IL_3_ have lower *H*_*R*32_, 2.19 and 3.27 MPa, respectively, signifying higher R-32 absorption than IL_1_. The process optimization suggests that both IL_2_ and IL_3_ can attain the minimum required purity. IL_2_ requires more process energy (193.95 kJ/kg) for R-410A separation than does IL_3_ (138.1 kJ/kg). This is primarily due to a higher R-125 absorption capacity of IL_2_ (*H*_*R*125_ = 6.72 MPa) compared to IL_3_ (*H*_*R*125_ = 19.49 MPa), leading to a smaller R-32 selectivity for IL_2_. This suggests that even ILs with very high selectivity (as in the case of IL_1_) can be infeasible/suboptimal. On the other hand, ILs with moderate selectivity can be feasible but may have high energy intensity.

Although IL_1_ has high R-32 selectivity, it has a smaller capacity, which makes it challenging to attain the required separation. Since our objective is to obtain energy-efficient ILs, it can be concluded that IL_2_ performs better than IL_1_ despite exhibiting lower R-32 selectivity. This analysis underscores two critical insights. First, achieving a sufficiently low *H*_*R*32_ (hence, high absorption capacity) is crucial for feasible process design. Second, relying solely on high selectivity can lead to infeasible/inferior solvent selection when evaluated against rigorous process performance criteria.

In this work, we consider a pool of 341,687 ILs and salts. These molecules are generated by taking cations and anions from a set of 683 cations and 505 anions in the COSMO-RS database. Figure 2a shows the distribution of the Henry’s constants across the entire data set. We observe many potential ILs that have a high R-32 solubility (*H*_*R*32_ ≤ 10 MPa) and low R-125 solubility (*H*_*R*125_ ≫ 10 MPa). Figure 2b shows the distribution of the R-32 selectivity. Many ILs have moderate to low R-32 selectivity (*S*_*R*32_ ≤ 5). Screening ILs solely based on selectivity is insufficient to ensure practical feasibility in refrigerant separation processes. We require the estimation of the infinite dilution activity coefficients as well as the solubility isotherms at multiple temperatures to estimate the binary interaction parameters for thermodynamic models.^20,30,31^ Evaluating these model parameters and performing exhaustive process optimization for all ILs is impractical. Clearly, it is nontrivial to identify the best ILs among the thousands of “*good*” candidates, which motivates the need for a framework capable of simultaneously addressing the minimum purity requirements (feasibility) and minimizing energy consumption (optimality) in R-410A separation.

**Figure 2:**
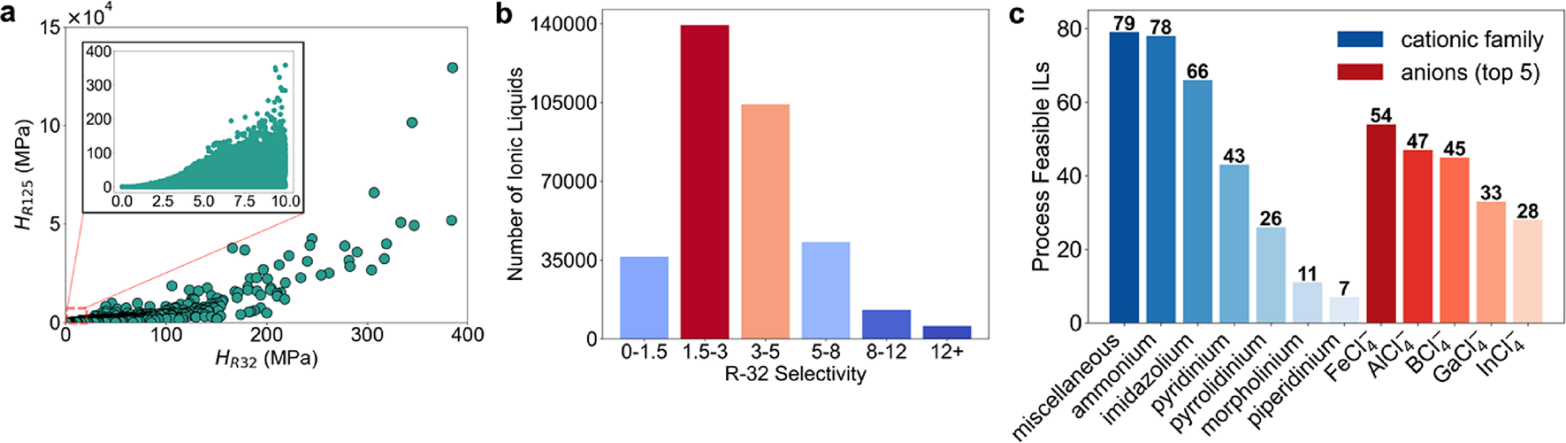
Mapping the solubility and selectivity of R-32 and R-125 in 341,687 ILs and salts. (a) COSMO-RS calculated Henry’s constants of R-32 and R-125. The inset shows those ILs with high R-32 absorption (*H*_*R*32_ ≤ 10 MPa). (b) Distribution of R-32 selectivity across the data set. (c) Distribution of process feasible ILs according to the major cationic families and anions.

## 3 Method

The computational workflow of our multiscale framework for the systematic screening of top-performing ILs for R-410A separation is shown in Figure 3. The framework includes five major steps. These are molecular structure generation, property prediction in COSMO-RS, solubility-based prescreening, rigorous process optimization and screening based on process performance, and postselection feasibility checks.

**Figure 3:**
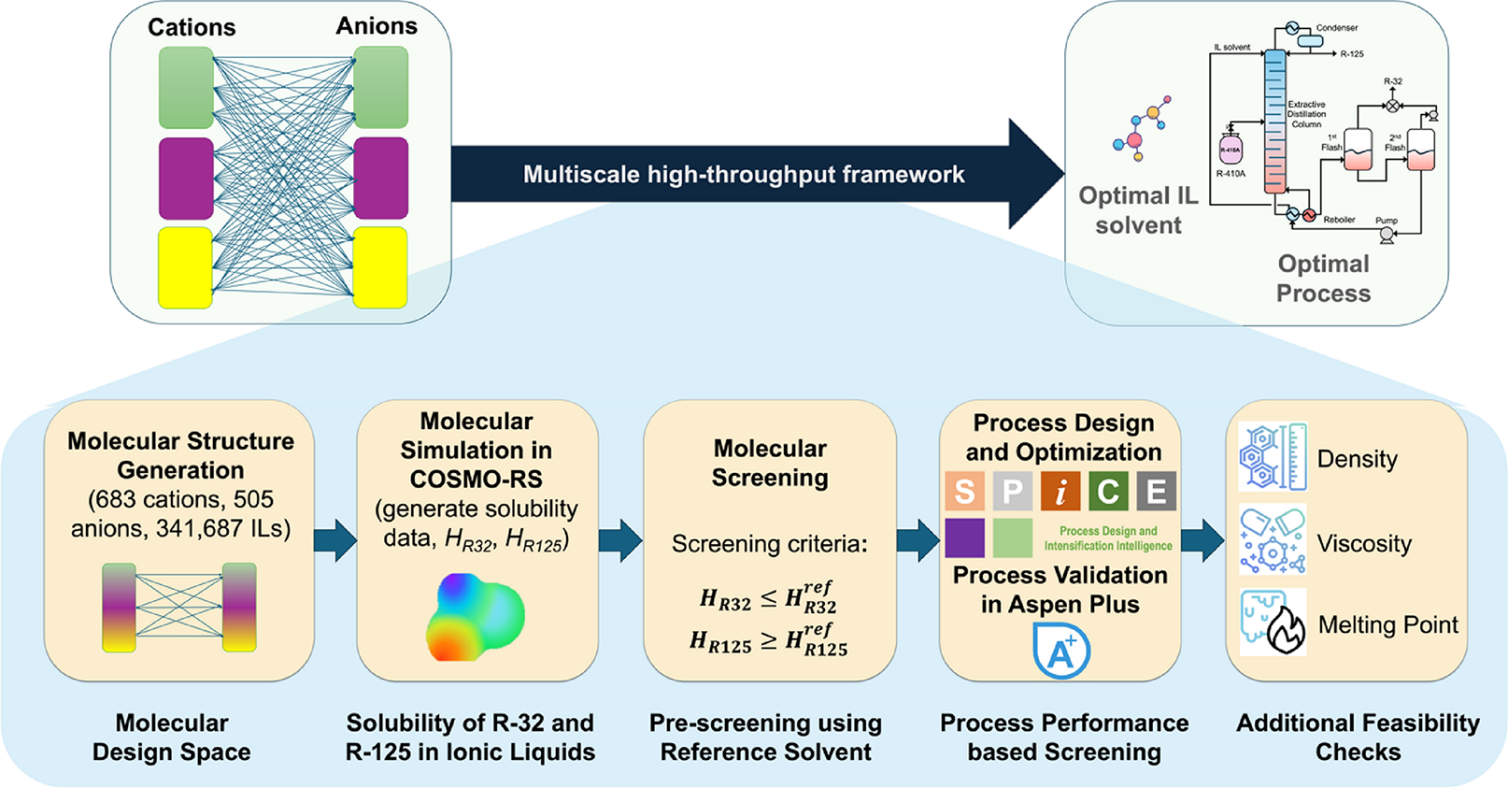
Multiscale high-throughput CAMPD framework for the systematic screening and process optimization of ILs for R-410A separation.

### 3.1 Molecular Structure Generation

We start with a superset of 683 cations and 505 anions. Each of these cationic and anionic species has a unit charge and is obtained from COSMOtherm. Any combination of these cations and anions produces an IL (or salt) with a neutral charge, totaling 341,687 unique molecular structures. The ILs and salts can be further categorized into six major cationic families. The distribution of ILs and salts in the major cationic families is given in Table 1. Full names of all cations and anions along with their structures are given in the Supporting Information. Imidazolium dominates the data set, providing 71,161 ILs and a wide range for R-32 selectivity. This is followed by pyridinium, ammonium, pyrrolidinium, morpholinium, and piperidinium. A miscellaneous group of 380 cations contributes the largest single share of ILs and salts. Within every family we observe ILs spanning low, moderate, and high R-32 selectivity.

**Table 1:** Number of Cations and ILs (or salts) in Major Cationic Families with the Maximum R-32 Selectivity

cation family	number of cations	number of ILs (or salts)	maximum R-32 selectivity
Imidazolium	143	71,161	37.05
Piperidinium	11	5512	12.76
Pyrrolidinium	19	9554	14.54
Morpholinium	9	4515	24.76
Pyridinium	67	33,092	19.01
Ammonium	54	26,978	32.91
Miscellaneous	380	190,875	551.84

### 3.2 Property Prediction

Recent work has shown that COSMO-RS-based solubility calculations of R-32 and R-125 capture the qualitative solubility trends well with experimental data.^25^ We collect experimentally measured Henry’s constants and compare them against COSMO-RS calculations. We observe that the COSMO-RS predictions qualitatively capture the trends of R-32 and R-125 solubility in ∼30 ILs at various temperatures (details are given in Section S7 of the Supporting Information). This suggests that the COSMO-RS-based solubility calculation is sufficient to provide a rank-ordered list of IL candidates for first-stage screening, as the top candidates are further evaluated through rigorous process optimization and feasibility checks (described later). This mitigates the risk of false positives. In this work, we leverage this predictive capability of COSMO-RS and estimate activity coefficients for all 341,687 ILs and salts. We generate the phase equilibria only for those ILs that are selected for process optimization. COSMO-RS predicts liquid-phase nonideality from quantum-chemical data. It has a two-step procedure. In the first step, every molecule (both refrigerants and IL) is assumed to be placed in an ideal conductor.^28^ The resulting screening charge on the surface is then sampled, and a histogram is constructed that depicts the area associated with each local charge density. This histogram is called the “sigma profile” of a molecule. The sigma profile, *P*(σ), essentially encodes the polarity landscape of the molecule. In the context of the IL-HFC system, the sigma profile of an IL solvent dictates its affinity toward polar or nonpolar surfaces of refrigerant components in a mixture. In the *C* + *A* approach,^29^ an IL is represented as an equimolar superposition of its cation and anion σ-profiles. This approach has been shown to reproduce experimental thermodynamic behavior across diverse IL families.^32^ In this work, we first extract σ-profiles for the cation and anion individually at the BP/TZVP-FINE level (COSMOtherm 2023) and then sum them point-wise to obtain a 50-bin descriptor for the ion-pair. No further geometry optimization is performed, because all conformers are retrieved from the preoptimized COSMObase library. The BP/TZVPD-FINE-23 parametrization improves the description of dispersion and hydrogen bonding relative to the original BP TZVP set.^33^

In the second step, we employ the statistical thermodynamics calculations in COSMO-RS to evaluate the excess chemical potential of a solute *k* at infinite dilution in solvent *s* by integrating pairwise surface contacts as follows:

$$\mu_{k}^{\mathrm{ex},\infty }=\int \int [E_{\mathrm{misfit}}(\sigma ,\sigma^{\prime })+E_{\mathrm{HB}}(\sigma ,\sigma^{\prime })+E_{\mathrm{vdW}}(\sigma ,\sigma^{\prime })]P_{k}(\sigma )P_{s}(\sigma^{\prime })d\sigma d\sigma^{\prime }$$ 1

where *E*_misfit_, *E*_HB*,*_ and *E*_vdW_ account for the electrostatic misfit interactions, hydrogen-bonding interactions, and van der Waals interactions, respectively.^28^ The excess chemical potential can then be used to compute the infinite dilution activity coefficients of the solute as follows:

$$\ln\;\gamma_{k}^{\infty }=\frac{\mu_{k}^{\mathrm{ex},\infty }}{k_{B}T}$$ 2

where *k*_B_ is the Boltzmann constant, and *T* is the system temperature. Next, we compute Henry’s constants and selectivity as follows:^34^

$$H_{i}=\gamma_{i}^{\infty }P_{i}^{s}$$ 3

$$S_{ij}={\left(\frac{H_{j}}{H_{i}}\right)}_{T}$$ 4

where γ_*i*_^∞^ is the infinite dilution activity coefficient of HFC solute *i* (R-32 and R-125) and *P*_*i*_^s^ is the saturation vapor pressure of solute *i* in an IL solvent. Selectivity of solute *i* over solute *j* is defined as the ratio of Henry’s constants of component *j* over component *i* in IL. Whenever needed, COSMO-RS is also used to compute the liquid–liquid equilibria. This is achieved by ensuring equal chemical potentials in the coexisting phases and minimizing the total Gibbs free energy of a mixture.

### 3.3 Solubility-Based Screening

A key concept in our framework is the use of a “reference” solvent. The currently known best solvent is considered as the reference solvent. Once a new IL emerges as the best solvent, we update the reference solubility parameters for the reference. This strategy enables the solubility parameters (i.e., Henry’s constants of R-32 and R-125) to serve as progressively tighter constraints for material-centric screening. This helps to discard many ILs in the screening step without having to perform computationally demanding process design and optimization calculations for all ILs.

For R-32 selective ILs, we require a low *H*_*R*32_ to facilitate high R-32 absorption. We also require a high *H*_*R*125_ to ensure low R-125 absorption. These two conditions provide the necessary solubility-based constraints to screen ILs as follows:

$$H_{R32}\leq H_{R32}^{\mathrm{ref}}$$ 5a

$$H_{R125}\geq H_{R125}^{\mathrm{ref}}$$ 5b

where *H*_*R*32_^ref^ and *H*_*R*125_^ref^ are the Henry’s constant values of the reference solvent. The top performing IL from our previous work^27^ provides the reference values. Specifically, a recently identified IL solvent 1-(2-hydroxyethyl)-3-methylimidazolium trifluoromethyltrifluoroborate has the following solubility parameters: Henry’s constant of R-32, *H*_*R*32_^ref^ = 3.27 MPa, and Henry’s constant of R-125, *H*_*R*125_^ref^ = 19.49 MPa, with an optimized process energy requirement of just 138.1 kJ/kg, the lowest value reported to date.^27^ Consequently, this IL is selected as the reference IL.

### 3.4 Process Performance-Based Screening

After the solubility-based prescreening, we obtain a reduced set of promising ILs as candidate solvents. For these ILs, we perform process optimization using SPICE, which is an in-house software tool for process synthesis and optimization.^13,35–37^ A description of the SPICE framework is provided in Section S1 of the Supporting Information. For a given set of thermophysical properties for an IL, SPICE can perform process synthesis and optimization under a set of prespecified objectives (e.g., minimization of the overall equivalent work for separation, minimization of the CO_2_ emission, or cost minimization).^38–40^ The output from SPICE is then used to determine the optimized process flowsheet with an extractive distillation column, two flash separators to regenerate ILs, and a recirculating pump to recycle the ILs back to the extractive distillation column. The key decision variables are (i) number and location of R-410A feed and IL entry stage, (ii) reflux ratio, (iii) optimal IL regeneration scheme, and (iv) flash temperatures and pressures.

In an extractive distillation process, as shown in Figure 1d, the IL is allowed to enter at the top stages, while R-410A is allowed to enter somewhere in the middle. In this work, the total number of stages in the extractive distillation column is fixed to 30. The side product stream(s) is allowed to withdraw from any stage. The R-32-rich IL is then regenerated using two flash separators in series. We also consider heat integration between the hot and cold streams, further reducing the process energy requirement. For process energy calculation, it is necessary to accurately predict the solubility behavior of both R-32 and R-125 in an IL. Many thermodynamic approaches exist for modeling the solubility behavior. These models range from simpler but computationally efficient Henry’s Law model (that provides acceptable predictions at dilute composition and low pressure regimes) to more complex cubic equation of state (EoS) models (e.g., Peng–Robinson^41^). In this work, we employ the Non-Random Two Liquid (NRTL) model (originally proposed by Renon and Prausnitz^42^ and modified for the special case of IL-HFC systems^31,43^ ) to characterize the solubility behavior of IL+R-410A in an extractive distillation column. Details on the NRTL model and its parameter estimation can be found in Section S2 of the Supporting Information. The process optimization provides two major information. First, the feasibility of an IL is checked, that is, whether HFC constituents can be recovered with ≥99.5 wt % purity. Once the minimum purity is met, the process operational variables are optimized to minimize the total equivalent work and CO_2_ emission required to separate R-410A.

### 3.5 Post-Selection Feasibility Checks

For the ILs that meet the minimum required purity, we perform postselection and in silico validation of the selected ILs. We check whether the set of ILs identified after process optimization satisfy major IL property limits. Specifically, we check the melting point (*T*_m_), density (ρ), and viscosity (η) of these ILs and enforce the following feasibility checks on these properties: (i) *T*_m_ ≤ 298.15 K, (ii) $\rho \leq 1600\;\frac{\mathrm{kg}}{m^{3}}$ at 298.15 K and 1 atm, (iii) η ≤ 100 mPa s at 298.15 K. We employ COSMOtherm to compute these properties, which uses quantitative structure property relationships (QSPR) that predict pure component properties from a given set of molecular descriptors. For example, IL density, ρ is computed as follows: $\rho =\frac{M_{w}}{\hat{V}N_{A}}$ , where *M*_w_ is the molecular weight of the IL, *V̂* is the corrected molar liquid volume of the IL, and *N*_A_ is Avogadro’s constant. *V̂* is obtained from a QSPR that requires seven descriptors and parameters (see Section 2.3.10 of the COSMOtherm reference manual^44^). The liquid viscosity at room temperature is also obtained from a five parameter QSPR model (see Section 2.3.11 of the COSMOtherm reference manual^44^). For the melting point, COSMOtherm uses a fast QSPR model (details can be found elsewhere^45^). We set the limits on density to be at 1600 kg m^–3^ at room temperature and atmospheric pressure because typical IL densities for imidazolium, pyridinium, and phosphonium ILs lie between 1000 and 1600 kg m^–3^.^46^ Regarding viscosity, a limit of 100 mPa s at 298.15 K has been applied in prior works for IL screening.^27,47^ Similarly, it is important to ensure that the selected IL remains liquid at room temperature. Thus, an upper bound of 298.15 K for the melting point is specified.

Our final selected ILs are those that (i) satisfy the solubility-based screening criteria, (ii) meet the minimum separation required in terms of R-32 and R-125 purity, and (iii) satisfy the specified property bounds on melting point, density, and viscosity.

## 4 Results and Discussion

Among the 341,687 molecules, only 355 ILs satisfy the solubility screening check (i.e., those that have *H*_*R*32_ values lower than 3.27 MPa and *H*_*R*125_ values higher than 19.49 MPa). The solubility-based screening allows us to discard ∼99% of ILs without having to perform full scale liquid–liquid equilibria (LLE) calculation and process optimization. A total of 310 ILs out of 355 ILs meet the minimum product purity requirement, which corresponds to a remarkable ∼90% success rate in identifying process-feasible ILs using the solubility-based screening criteria (eqs 5a and 5b). These 310 feasible ILs are listed in Table S1 of the Supporting Information. The optimized NRTL parameters and complete process optimization results for all 310 ILs are also given in the Supporting Information.

Figure 2c shows the distribution of 310 feasible ILs across each of the major cationic families. Among them, the ammonium, imidazolium, and pyridinium-based IL families constitute more than 60% of the total feasible ILs found. [FeCl_4_]^−^ appears to be the top anion that is present in 54 of the 310 feasible ILs. Interestingly, all five top anions contain chloride. Figure 4a shows the process energy consumption of the 310 ILs that were found to be feasible, along with four of the well-known existing ILs and the reference IL (IL_3_). Process optimization results for the four existing ILs are given in Section S4 of the Supporting Information. The process energy consumption decreases with an increase in the R-32 selectivity. Interestingly, among the newly identified ILs, there are multiple ILs with the same R-32 selectivity but different process energy requirements. Figure 4b shows the relative solubility of selected ILs in the entire solubility space considered. The newly identified ILs show a slightly lower R-32 absorption capacity than the four existing ILs. However, the R-125 absorption is much lower in these newly identified ILs compared to that of the existing ILs. Out of the 310 ILs that meet the minimum purity, 285 ILs require lower process energy than [EMIM]­[SCN], which is a well-known IL for R-410A separation.^47^ The top 10 selected ILs are shown in Figure 4c. The top IL identified is 1-(3-methoxypropyl)-1-methylpyrrolidinium tetrachloroferrate ([MOPMPy]­[FeCl_4_]) with a separation process energy requirement of 124.13 kJ/kg. This represents a 13.30% reduction in process energy consumption compared to the best existing IL [EMIM]­[SCN][Bibr ref47] and a 10.12% reduction in process energy consumption compared to the reference IL 1-(2-hydroxyethyl)-3-methylimidazolium trifluoromethyltrifluoroborate.^27^

**Figure 4:**
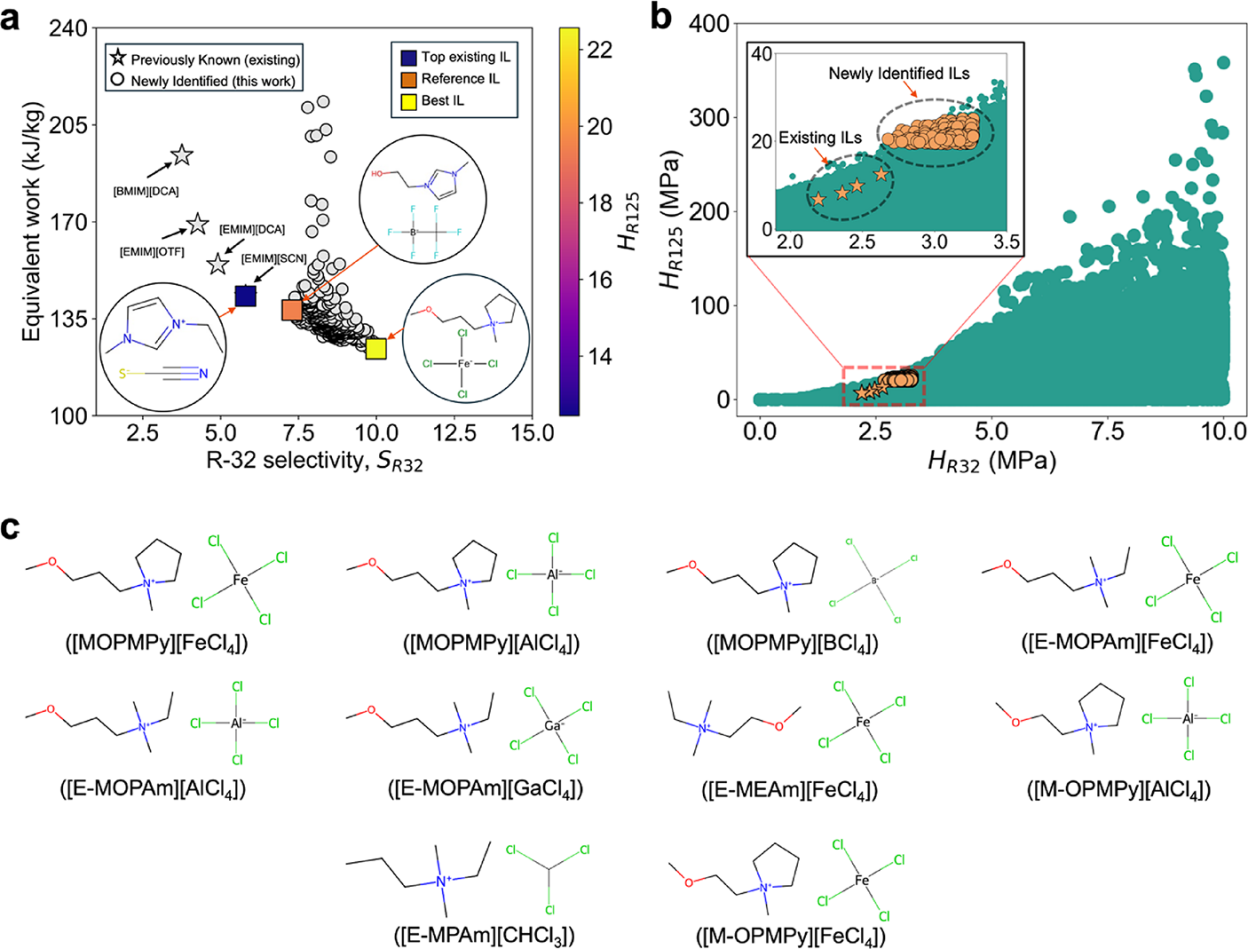
Comparison of process-feasible ILs and existing ILs in terms of energy intensity and solubility. (a) Equivalent work for separation vs R-32 selectivity, (b) Henry’s constants of R-32 and R-125, and (c) molecular structures and acronyms of the top ten selected ILs based on the lowest process energy consumption. Full names of the top ten selected ILs are given in Table S3 of the Supporting Information.

The top five selected ILs from each of the six major cationic families are listed in Table 2, along with their properties and process performance values. We observe consistent anion motifs. Tetrachlorometallate anions (FeCl_4_^–^, AlCl_4_^–^, GaCl_4_^–^, or BCl_4_^–^) appear in 18 of the 25 selected ILs. For these ILs, *H*_*R*125_ > 20 MPa, while *H*_*R*32_ ranges between 2.7 and 3.3 MPa. This results in the desired R-32 and R-125 separation characteristic. We also observe that methoxy-alkyl side chains dominate the cation space. Most top cations contain a 2- or 3-methoxyethyl/propyl substituent. Short ether chains appear to increase R-32 solubility (lower *H*_*R*32_) while maintaining a moderate molecular weight and viscosity. All families except morpholinium demonstrate a typical viscosity between 18–35 mPa s, which is far lower than the imposed constraint of 100 mPa s. Overall, pyrrolidinium and ammonium ILs exhibit energy demand between 124 to 127 kJ/kg with viscosities below 30 mPa s, suggesting favorable ILs.

**Table 2:** Top ILs from Each Cationic Family^a^

		*M*_w_	*H*_*R*32_	*H*_*R*125_	viscosity	density	melting point	eq work
cation	anion	(g/mol)	(MPa)	(MPa)	(cP)	(g/cm^3^)	(K)	(kJ/kg)
1-(3-methoxypropyl)-1-methylpyrrolidinium	tetrachloroferrate(iii)-hextuplet	355.92	2.77	22.57	26.56	1.356	268	124.14
1-(3-methoxypropyl)-1-methylpyrrolidinium	tetrachloroaluminate	327.06	2.81	22.30	27.97	1.240	266	124.41
1-(3-methoxypropyl)-1-methylpyrrolidinium	bcl4	310.89	2.90	22.51	35.72	1.232	251	125.41
1-(2-methoxyethyl)-1-methylpyrrolidiniu m	tetrachloroaluminate	313.03	3.00	23.56	20.55	1.272	265	127.05
1-(2-methoxyethyl)-1-methylpyrrolidinium	tetrachloroferrate(iii)-hextuplet	341.89	2.96	23.53	19.52	1.396	268	127.24
ethyl-(3-methoxypropyl)-dimethylammonium	tetrachloroferrate(iii)-hextuplet	343.91	2.88	23.49	27.44	1.338	281	125.97
ethyl-(3-methoxypropyl)-dimethylammonium	tetrachloroaluminate	315.05	2.93	23.49	28.90	1.220	278	125.98
ethyl-(3-methoxypropyl)-dimethylammonium	tetrachlorogallate	357.80	2.86	22.14	28.90	1.402	280	126.78
ethyl-dimethyl-2-methoxyethylammonium	tetrachloroferrate(iii)-hextuplet	329.88	3.07	24.72	20.01	1.377	282	126.96
ethyl-dimethyl-propylammonium	chcl3	234.59	3.20	23.11	26.15	1.151	297	127.12
1-(ethoxymethyl)-3-methyl-imidazolium	tetrachloroferrate(iii)-hextuplet	338.85	3.02	22.84	26.65	1.462	263	127.74
1-(2-methoxyethyl)-3-methylimidazolium	tetrachloroferrate(iii)-hextuplet	338.85	3.01	22.73	18.42	1.463	263	127.88
1-ethyl-2,3-dimethyl-imidazolium	chcl3	243.56	3.07	21.23	19.04	1.245	295	128.85
1-(2-methoxyethyl)-3-methylimidazolium	tetrachlorogallate	352.74	3.00	21.73	19.39	1.534	263	129.53
1-ethyl-3-methyl-imidazolium	tetrachloroferrate(iii)-hextuplet	308.83	3.21	24.56	12.64	1.506	272	129.70
1-(ethoxymethyl)pyridinium	chcl3	256.56	3.26	23.62	33.21	1.273	256	128.13
1-propylpyridinium	chcl3	240.56	3.22	22.56	23.00	1.253	265	129.13
1-(3-methoxypropyl)pyridinium	tetrachloroaluminate	321.01	2.92	20.86	26.36	1.302	252	129.65
1-(3-methoxypropyl)pyridinium	bcl4	304.84	3.07	22.08	33.55	1.296	238	129.76
1-(2-methoxyethyl)pyridinium	tetrachloroaluminate	306.98	3.20	24.50	18.85	1.344	252	129.76
1-(3-methoxypropyl)-1-methylpiperidinium	tetrachloroaluminate	341.08	2.71	20.20	29.44	1.229	270	129.82
1-(2-methoxyethyl)-1-methylpiperidinium	tetrachloroaluminate	327.06	2.88	20.56	21.92	1.258	271	130.42
1-(3-methoxypropyl)-1-methylpiperidinium	tetrachloroferrate(iii)-hextuplet	369.95	2.68	20.48	27.93	1.339	273	132.14
1-(3-methoxypropyl)-1-methylpiperidinium	bcl4	324.91	2.79	20.16	37.70	1.221	256	132.97
1-(2-methoxyethyl)-1-methylpiperidinium	bcl4	310.89	2.98	21.15	27.97	1.250	256	138.68
4-(2-methoxyethyl)-4-methylmorpholinium	bclf3	263.50	3.23	23.27	53.81	1.262	178	130.00
4-(2-methoxyethyl)-4-methylmorpholinium	asf6	349.15	3.20	22.35	47.44	1.597	169	132.94
4-(ethoxymethyl)-4-methylmorpholinium	bclf3	263.50	3.16	20.97	58.54	1.261	179.49	133.77
4-(2-ethoxyethyl)-4-methylmorpholinium	tetrachloroindium	430.89	3.19	24.28	46.75	1.610	272.08	134.92
4-(ethoxymethyl)-4-methylmorpholinium	asf6	349.15	3.11	20.18	51.61	1.597	170.52	137.33
ethyl-dimethylsulfonium	bf4	178.01	3.15	23.02	99.09	1.313	293	127.38
triethylsulfonium	chcl3	237.62	3.19	22.65	22.39	1.212	367	127.82
n,n,n’,n’-tetramethyl-1,2-ethanediamine	bcl4	269.84	3.16	22.53	23.16	1.234	273	128.50
n,n,n’,n’-tetramethyl-1,2-ethanediamine	tetrachloroaluminate	286.01	3.05	21.30	18.28	1.243	288	129.43
triethylsulfonium	tetrachloroferrate(iii)-hextuplet	316.91	3.16	22.88	14.81	1.423	342	129.73

a Molecular weight, Henry’s constants, and pure component property values are extracted from COSMO-RS. Optimized equivalent work is obtained from SPICE.

We compare the optimized process flowsheets for [EMIM]­[SCN] and [MOPMPy]­[FeCl_4_] in Figure 5a,b. For the same feed pressure of 10 bar, the same operating pressure of the extractive distillation column, and the same feed flow rate of 10 kg/h, significant differences in optimal operating conditions are observed for these two ILs. Specifically, [MOPMPy]­[FeCl_4_]-based process operates at a reduced reflux ratio (1.4 vs 2.3) and exhibits lower operational temperature (314 K vs 320 K) in the primary flash regeneration unit (Flash 1) relative to the process that uses [EMIM]­[SCN] as solvent. The R-410A feed enters the extractive distillation column at varying stages (stage 24 for [EMIM]­[SCN] vs stage 26 for [MOPMPy]­[FeCl_4_]). In both processes, the IL solvent is introduced at the second stage of the extractive distillation column. A lower reflux ratio results in less R-32 and R-125 circulation inside the distillation column, which reduces both the condenser and reboiler duties.

**Figure 5:**
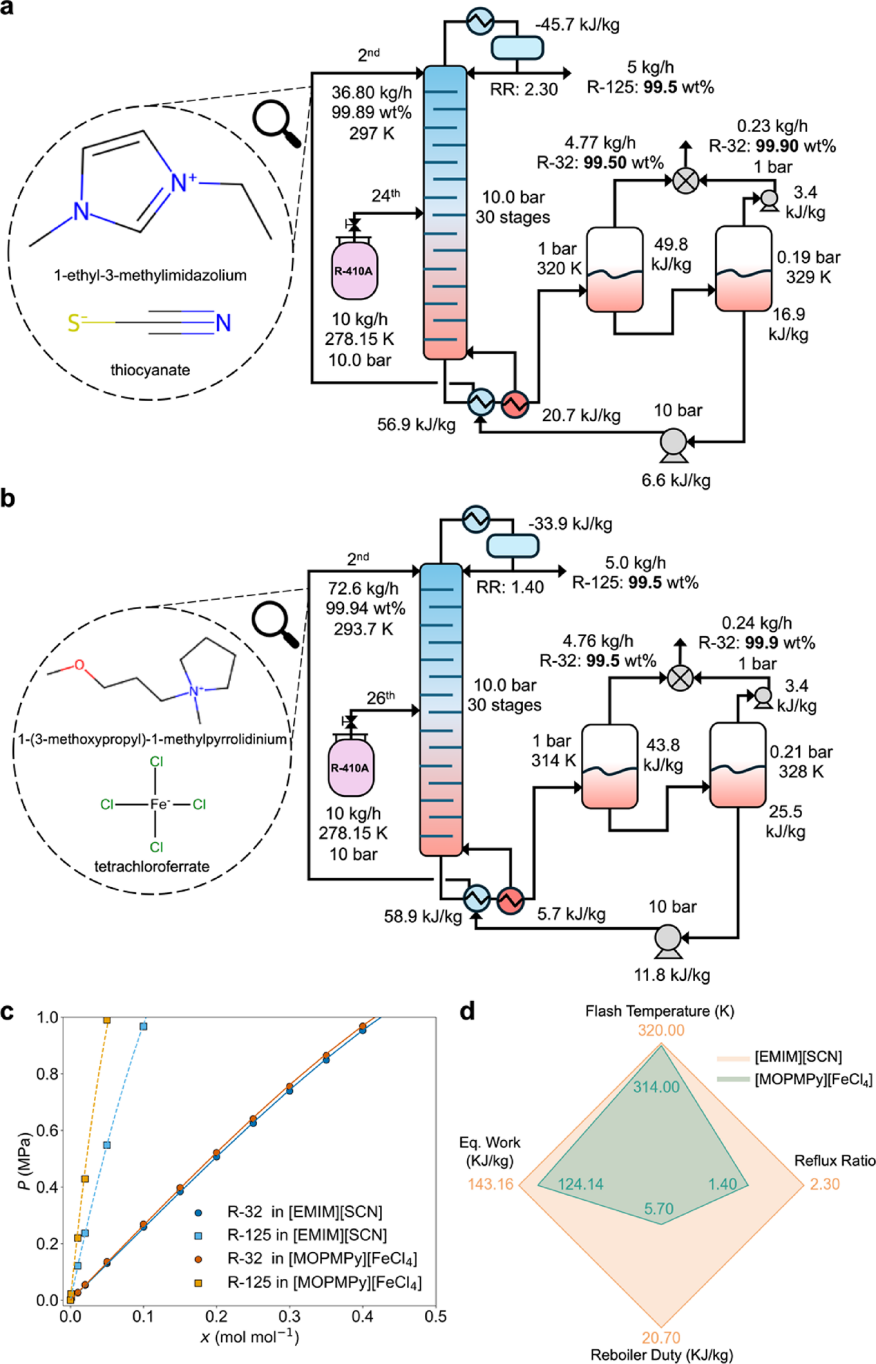
Optimized process flowsheets for (a) [EMIM]­[SCN] and (b) [MOPMPy]­[FeCl_4_] solvents. (c) Solubility of R-32 and R-125 in [EMIM]­[SCN] and [MOPMPy]­[FeCl_4_] at 298.15 K. Here, the equilibrium data are estimated using COSMO-RS, which are shown as square data points. The solid and dashed lines depict the fitted NRTL model prediction for R-32 and R-125 in ILs, respectively (for details about NRTL fitting, see Section S2 of the Supporting Information). (d) Comparison of key process conditions between [EMIM]­[SCN] and [MOPMPy]­[FeCl_4_]-based extractive distillation processes.

To understand the solubility behavior of the top performing ILs, we compare the solubility isotherms (P-x diagram) of [EMIM]­[SCN] and the top IL [MOPMPy]­[FeCl_4_] at 298.15 K, as shown in Figure 5c. A P-x diagram shows the equilibrium absorption of solutes in solvents at different pressures and temperatures. Both ILs demonstrate almost identical R-32 absorption characteristics, but [MOPMPy]­[FeCl_4_] exhibits a significantly lower R-125 absorption capacity than [EMIM]­[SCN]. The larger difference in the absorption of R-32 and R-125 helps the extractive distillation process to achieve the product purity at a relatively lower reflux ratio and lower regeneration temperature. At the process level, this translates to the requirement of a lower energy consumption in the reboiler and lower cooling duty for the condenser. The major differences in process operating conditions that lead to the superior performance of [MOPMPy]­[FeCl_4_] over [EMIM]­[SCN] are shown in Figure 5d.

There appear to be many ILs with very high R-32 selectivity (see Figure 2a). However, the top selected ILs and their properties indicate that just screening based on the highest selectivity is indeed problematic, as feasibility in the process operation is not always attained. Also, the same selectivity can be obtained by multiple combinations of the Henry’s constants, making it hard to make decisions based on selectivity alone. This leads to the additional screening requirement based on the absorption capacity of R-32. While some selectivity is important for azeotropic separation, selectivity should be used in tandem with the absolute solubility, as indicated by the inverse of Henry’s constant, and thus must be looked at from the point of view of a reference solvent. We need ILs to attain the minimum required purity for feasible process operation. For this, we need low *H*_*R*32_ and high *H*_*R*125_. Once the feasibility condition is met, only then, with increasing selectivity, do we observe a decrease in the process energy consumption (Figure 4a).

Four out of the top 10 ILs identified in this study contain an iron-based (FeCl_4_^–^) anion. To better understand the favorable molecular performance of these anions in selected ILs, we study the distribution of surface charge densities, described by the sigma profiles of the top 10 best-performing ILs and HFC mixture constituents (R-32 and R-125) (see Figure 6a). Sigma profiles provide insights into the intermolecular forces that influence the solubility of refrigerants. Both R-32 and R-125 are fluorinated organic molecules; however, R-32 contains more electropositive hydrogen atoms than R-125. Consequently, R-32 generally exhibits a higher solubility in ILs compared to R-125. Nonetheless, R-125 typically displays greater sensitivity to variations in IL solvents.^12,25^ Both refrigerants (R-32 and R-125) exhibit a maximum in the nonpolar region (−0.01 ≤ σ ≤ +0.01 eÅ^–2^). This confirms that dispersion (van der Waals) forces are the principal drivers of absorption in ILs.^12,25,48^ R-125 is almost completely fluorinated. Therefore, its surface is skewed toward slightly negative σ values (electron-rich). A modest shoulder at σ ≈ −0.01 eÅ^–2^ arises from the single C–H bond of R-125 in the −CHF_2_ group, where the hydrogen is only weakly electropositive because of the strong effect of neighboring F atoms. The top-performing ILs (colored curves) achieve this with a characteristic “double-humped” profile where twin peaks flank a trough centered at around σ ≈ 0 eÅ^–2^. The peaks align with the maximum of R-32, whereas the trough overlaps with the central maximum of R-125, thereby penalizing its uptake. We observe that halometallate anions such as [FeCl_4_]^−^, [AlCl_4_]^−^, and [GaCl_4_]^−^ create exactly this pattern. Regarding cation effects, increasing alkyl chain length enhances the solubility of both R-32 and R-125 in ILs, although the effect is more pronounced for R-125.^41^ The presence of extensive nonpolar regions from longer alkyl chains appears to stabilize R-125 by reducing the electrostatic repulsions. Thus, shorter alkyl chains are generally preferable for refrigerant separation, since longer chains disproportionately enhance R-125 solubility. We observe a similar trend in all the top five ILs for each cationic family, which are shown in Figure S4 of the Supporting Information.

**Figure 6:**
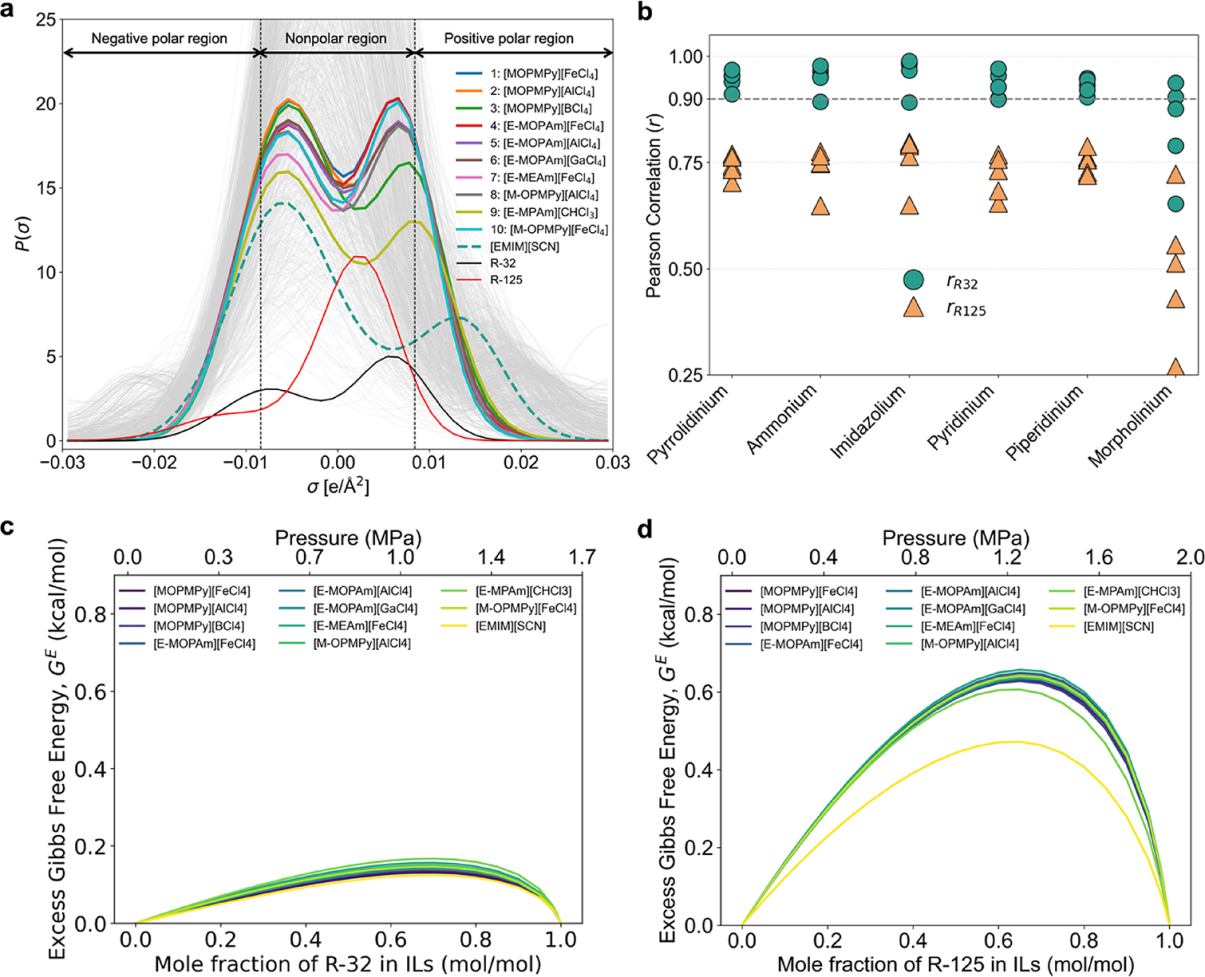
Understanding the molecular features of top performing ILs. (a) Sigma profiles of the top 10 process feasible ILs. Randomly selected sigma profiles of 1000 ILs from the 341,689 ILs are shown in light gray. Here, sigma profiles of the entire IL are obtained by the C+A ion-pair profiles where individual cation and anion profiles are summed pointwise. (b) Pearson correlation of sigma profiles between the top 5 ILs from each cationic family with R-32 and R-125. (c) Variation of excess Gibbs free energy with R-32 compositions. (d) Variation of excess Gibbs free energy with R-125 compositions.

Designing an IL that prefers R-32 over R-125 thus amounts to providing surface segments that coincide with the R-32 maximum inside the nonpolar region and avoid σ ranges where R-125 concentrates. To that end, we quantitatively assess the similarity between the sigma profiles of ILs and reference refrigerants (R-32 and R-125). We employ the Pearson correlation, which allows for a rigorous comparison of the charge density distributions to capture structural alignment. The Pearson correlation coefficient (*r*) measures the linear relationship between two sigma profiles, evaluating how well the fluctuations in one profile correspond to those in the other. This is computed as follows:

$$r=\frac{\sum_{i=1}^{n}(X_{i}-\overset{-}{X})(Y_{i}-\overset{-}{Y})}{\sqrt{\sum_{i=1}^{n}(X_{i}-\overset{-}{X}{)}^{2}}\sqrt{\sum_{i=1}^{n}(Y_{i}-\overset{-}{Y}{)}^{2}}}$$ 6

where X = [*X*_1_, *X*_2_, ···, *X*_*n*_] and *Y* = [*Y*_1_, *Y*_2_, ···, *Y*_*n*_] represent the sigma profiles of the IL and the reference refrigerant (R-32 or R-125), respectively. *X̅* and *Y̅* denote their mean values. The correlation coefficient *r* ranges from −1 to 1, where *r* = 1 indicates perfect positive correlation (identical shape and trend), *r* = −1 indicates perfect negative correlation (opposite peaks and trends), and *r* = 0 implies no correlation. In the context of our work, a high Pearson correlation signifies that the IL has a sigma profile similar to that of the reference refrigerant. A lower or negative Pearson correlation suggests deviations in the charge density distribution. We compute the Pearson correlation for the top five ILs in all of the major cationic families. Results are given in Figure 6b. In all ILs, except for those from the morpholinium family, we observe a high Pearson correlation (∼0.9) between the sigma profiles of ILs and R-32, and a low Pearson correlation between the sigma profiles of ILs and R-125. In all ILs, we observe *r*_*R*32_ ≫ *r*_*R*125_, validating the hypothesis that the sigma profiles of a “good” IL solvent are highly similar to those of the sigma profile of R-32 (to favor the uptake) and need to show opposite peaks in the nonpolar region for R-125 (to penalize the uptake). This understanding can act as a design rule for future IL-based solvent design. While Pearson correlation is a helpful metric, it is not universally predictive; notable exceptions are discussed in Section S6 of the Supporting Information.

The gas solubility is also closely linked to the excess Gibbs free energy (*G* ^*E*^) of the mixing. The *G* ^*E*^ of R-125 in an IL should be higher to discourage absorption. For R-32, it needs to be lower to facilitate better absorption. Figure 6c,d show the *G* ^*E*^ for the top 10 ILs in R-32 and R-125, respectively. For R-32, all these ILs depict near-zero values, suggesting near-spontaneous absorption of R-32. Compared to [EMIM]­[SCN], the top ILs have higher peaks for R-125, suggesting a greater repulsion toward R-125. This further rationalizes the selection of the top ILs in terms of their superior performance.

To summarize, while solubility and selectivity are established molecular-level metrics, our work embeds these metrics in a multiscale solvent-to-process flowsheet workflow and applies a rigorous process-level optimization to each IL candidate, ultimately extracting design rules that link sigma profile features to both molecular and process performance. This holistic approach, through the introduction of a “reference IL”-based approach, prunes 99% of the initial search space and identifies 285 ILs that outperform the benchmark [EMIM]­[SCN] on equivalent work. The top performing ILs reveal the dominance of Cl-rich tetrachlorometallate anions. These insights are observed based on process performance and are not attainable from solubility data alone.

## 5 Conclusions

We presented a multiscale framework for the selection of the optimal IL in an extractive distillation process for azeotropic R-410A refrigerant separation. By integrating molecular simulation and solubility-based screening with rigorous process optimization, our methodology was able to identify many ILs, indicating a superior separation performance. Starting with a design space of 341,687 ionic liquids and salts, we identified solvent candidates that are simultaneously thermodynamically attractive, process energy-wise less demanding, and operationally viable. A “reference” IL-based approach helped prune ∼99% of the design space and required rigorous process calculations for only 355 candidates. In doing so, we eliminated the need for exhaustive process optimization for all 341,687 candidates. Among the 355 IL candidates, 310 ILs ultimately met the required purity of recovered HFCs (≥99.5 wt %). The workflow revealed 285 previously unexplored ionic liquids that surpass the energy performance of the current benchmark, [EMIM]­[SCN]. The top performing IL, namely, 1-(3-methoxypropyl)-1-methylpyrrolidinium tetrachloroferrate­(III) or [MOPMPy]­[FeCl_4_], reduces the equivalent work of R-410A separation to 124 kJ/kg, which represents a 13.30% improvement over the energy requirement for [EMIM]­[SCN]. New insights into the IL chemical design space reveal critical structural factors that influence selectivity toward specific refrigerant components (R-32 or R-125). Notably, the presence of more H atoms in R-32 than in R-125 facilitates stronger attraction to an IL, thereby making ILs more probable to be R-32 selective. Systematic inspection of the sigma profiles of the top performing ILs reveals a chloride-rich tetrachlorometallates (e.g., FeCl_4_^–^ and AlCl_4_^–^) with weakly electronegative charge distribution as attractive anions. Also, short methoxy-substituted side chains on pyrrolidinium or ammonium cations enhance R-32 absorption with moderate viscosity. Solubility-based screening and process optimization reveal that we need a favorable combination of Henry’s constants to first obtain an IL with feasible process operation, and just screening ILs based on the highest selectivity may not always achieve a feasible separation process. If an IL does not sufficiently absorb R-32, then the process fails to perform regardless of how high the R-32 selectivity is. The immediate next step is to experimentally synthesize and measure the solubility of R-32 and R-125 in the most promising ILs identified by our framework. Future work could include toxicity and life-cycle assessments in the framework, thereby providing a more holistic screening of IL candidates. Physics-informed hybrid modeling techniques^49^ can be applied to increase the accuracy and consistency of material and process property models. The Supporting Information includes a ranked list of 285 IL candidates that are predicted to require less process work than [EMIM]­[SCN]. This list may provide a pool of potentially more sustainable IL solvents for future experimental validation and life-cycle assessment studies. The framework is readily applicable to other azeotropic or close-boiling separations, given that appropriate thermodynamic models and data are available. Overall, it highlights how the integration of molecular simulation, solubility-based screening, and process optimization can convert an otherwise intractable combinatorial problem into a data-driven tractable problem to discover new IL solvents for HFC reclamation.

## Supplementary Material





## Notes

Optimization of the extractive distillation process; details of the thermodynamic modeling; list of all feasible ionic liquids; process performance of existing ionic liquids; details of sigma profiles and excess Gibbs free energy analysis; additional analysis of Pearson correlation of sigma profiles of process feasible and infeasible ionic liquids; comparison of COSMO predicted Henry’s constants with experimental data, and full name and structure of all cations and anions (PDF)
Anion and cation images (ZIP)

## §

The Dow Chemical Company, Freeport, Texas, United States
