# Supplementary material for: Integrated Molecular Screening and Process Optimization To Identify Ionic Liquids for Energy-Efficient Refrigerant Separation
Source: ACS Sustain Chem Eng. 2025 Sep 10;13(37):15435–46. doi: 10.1021/acssuschemeng.5c05520 (PMC12458977; doi:10.1021/acssuschemeng.5c05520)
Supplement: Supplementary file 1 [file sc5c05520_si_001.pdf]

Supporting Information

Integrated Molecular Screening and Process Optimization to Identify Ionic Liquids for Energy-Efficient Refrigerant Separation

Ashfaq Iftakher[1], Mohammed Sadaf Monjur[1,†], Ahaduzzaman Nahid[1], Michael Dukissis[1] and M. M. Faruque Hasan[1,2,*]

[1]Artie McFerrin Department of Chemical Engineering, Texas A&M University, College Station, TX 77843-3122, USA.
[2]Texas A&M Energy Institute, Texas A&M University, College Station, TX, 77843, USA.

This file includes:

Number of pages: 27

Number of figures: 7

Number of tables: 4

S1

*Correspondence should be addressed to M.M. Faruque Hasan at hasan@tamu.edu, Phone: (979) 862-1449.
†Mohammed Sadaf Monjur is currently with The Dow Chemical Company, Freeport, TX, United States

S1. Optimization of the Extractive Distillation Process

We optimize the extractive distillation process using the SPICE_ED (Synthesis and Process Intensification of Chemical Enterprises Extractive Distillation) framework(Monjur et al., 2022). This is an advanced computational platform for systematic design and optimization of extractive distillation processes, particularly for separating azeotropic mixtures like high-GWP refrigerants using ionic liquids (ILs) as solvents. Conventional methods often rely on fixed flowsheets and expert-driven trial-and-error, but SPICE-ED eliminates this limitation through a flexible, bottom-up modeling strategy. It uses a building block-based representation(Demirel et al., 2017) to capture the fundamental physicochemical phenomena of separation processes. Each building block represents a process unit or interface, such as mixing, vapor-liquid equilibrium (VLE), or heat exchange, and the blocks are assembled into a superstructure that encodes all possible process configurations. The SPICE_ED framework is built on a rigorous set of mass and energy balance equations, phase equilibrium relationships, and thermodynamic property models. These equations are essential for accurately representing the performance of extractive distillation processes and are embedded into the optimization model for flowsheet synthesis. This formulation is translated into a Mixed-Integer Nonlinear Programming (MINLP) problem and solved using global optimization algorithms to identify optimal designs based on selected criteria such as minimum energy use, separation cost, or CO_2-equivalent emissions. Unlike traditional tools like Aspen Plus that require a predefined flowsheet, SPICE_ED explores all feasible alternatives automatically, enabling true process intensification. Moreover, SPICE_ED includes rigorous thermodynamic models such as Gamma–Phi(Shiflett et al., 2006) for refrigerant/IL solubility and offers surrogate-based property estimation(Iftakher et al., 2022) to maintain computational tractability. The framework has been validated against commercial simulation results, confirming its reliability for real-world applications.

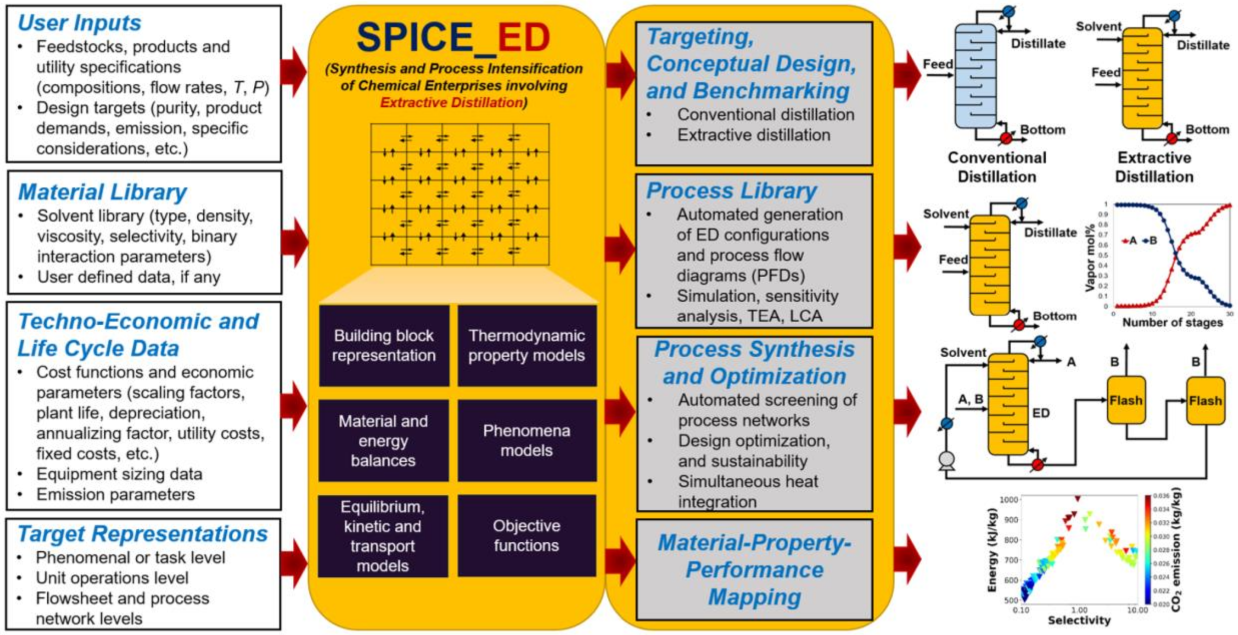

Figure S1: The SPICE_ED framework. Adapted with permission from (Monjur et al., 2022). Copyright (2022) American Chemical Society.

Figure 1 illustrates the SPICE_ED framework, which is built upon a building block-based process representation. This approach allows the representation of extractive distillation processes from the fundamental phenomena level, enabling innovative designs without the need for predefining unit operations or flowsheets. In the early design phase, SPICE_ED can assess whether extractive distillation is a viable intensification strategy for a specific separation task, based on thermodynamic feasibility and process conditions. It also facilitates conceptual design. Users can input specifications like feed composition, desired product purities, solvent properties, and utility constraints to evaluate the potential of various process configurations before committing to detailed design. SPICE_ED enables detailed process simulation and analysis. With fixed design parameters, it can simulate performance metrics such as energy consumption, CO_2 emissions, and material recovery rates using rigorous thermodynamic and transport models. The process synthesis and optimization capability is a central feature of the framework. By formulating a superstructure-based MINLP problem, SPICE-ED can automatically generate, evaluate, and select optimal designs based on objectives such as energy efficiency, sustainability, or cost minimization.

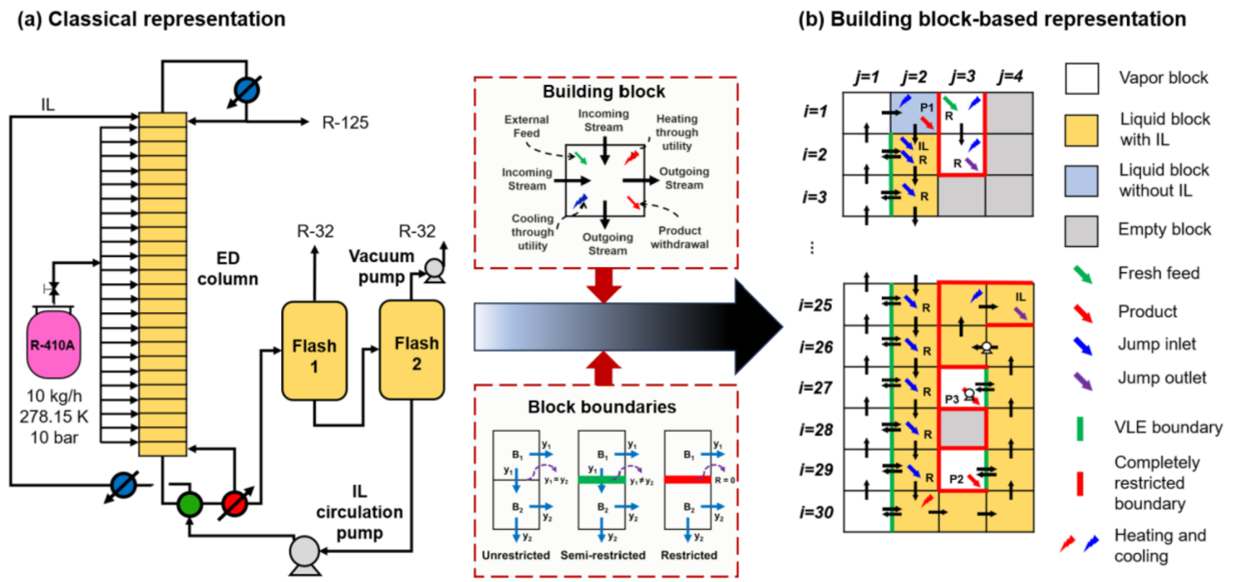

Figure S2: Transformation of a classical extractive-distillation process flowsheet to an equivalent building block-based representation. Adapted with permission from (Iftakher et al., 2025). Copyright (2025) Elsevier.

Figure S2 provides a equivalent transformation between the classical and the building block-based representations of the extractive distillation process for separating R-410A into its components, R-32 and R-125, using ILs. On the left (Figure S2(a)), the classical representation illustrates the flowsheet of a solvent-based separation process. R-410A enters the extractive distillation column, where IL is fed from the top and the refrigerant mixture enters at a designated tray. Inside the column, R-125 is recovered as the distillate, while R-32, being more soluble in IL, exits with the IL from the bottom. This mixture is then sent through two flash separators, Flash 1 and Flash 2, where R-32 is progressively separated and recovered. The regenerated IL is recycled back to the column via an IL circulation pump. On the right (Figure S2 (b)), the building block-based representation transforms the process into a modular, grid-based superstructure suitable for systematic optimization using the SPICE-ED framework. Here, the entire process is discretized

into a 30 × 4 grid where each block can perform a specific function (e.g., vapor or liquid phase handling, phase change, heating, cooling, or chemical separation). Each block interacts with its neighbors through "block boundaries" that can be unrestricted, semi-restricted, or completely restricted, depending on the physical interactions (e.g., flow continuity or phase equilibrium). This representation includes various process features: fresh feed (green arrows), product withdrawal (red arrows), jump streams (blue and purple arrows indicating material routing across non-adjacent blocks), and heat integration (red-blue arrows for heating and cooling). This modular representation enables rigorous optimization by treating the configuration and operating conditions as decision variables. It allows the solver to explore a vast design space to identify energy-efficient and operationally feasible configurations.

S2. Thermodynamic Modeling

Vapor-liquid equilibrium (VLE) for component k in an N component mixture is written as(Shiflett et al., 2006):

$$y_k P \Phi_k = x_k \gamma_k P^s, \text{ for } k = 1, \dots, N \tag{S1}$$

where x_k and y_k are the liquid- and vapor-phase equilibrium mole fractions, γ_k is the liquid-phase activity coefficient, Φ_k is the vapor-phase fugacity coefficient, and P^s_k is the saturation pressure of component k at temperature T.

The saturation pressure is calculated using the Antoine expression:

$$\ln P_k^s = A_k^s - \frac{B_k^s}{T + C_k^s} \tag{S2}$$

with A_k^s, B_k^s and C_k^s the Antoine parameters for component k.

The vapor phase fugacity coefficient is estimated by:

$$\Phi_k = \exp\left(\frac{(B_k - V_K)(P - P_k^s)}{RT}\right) \tag{S3}$$

where B_k is the second virial coefficient ($\frac{cm^3}{mol}$) and V_k is the saturated molar volume of component k.

The activity coefficient in the liquid phase is calculated as follows:

$$\ln \gamma_1 = x_2^2 \left[\tau_{21}\left(\frac{G_{21}}{x_1 + x_2 G_{21}}\right)^2 + \left(\frac{\tau_{12} G_{12}}{(x_2 + x_1 G_{12})^2}\right)^2\right] \tag{S4}$$

where τ_{12} and τ_{21} defined as follows:

$$\tau_{12} = \tau_{12}^{(0)} + \frac{\tau_{12}^{(1)}}{T} \quad \text{(S5)}$$

$$\tau_{21} = \tau_{21}^{(0)} + \frac{\tau_{21}^{(1)}}{T} \quad \text{(S6)}$$

The four τ parameters ($\tau_{12}^0, \tau_{12}^1, \tau_{21}^0, \tau_{21}^1$) are fitted to the experimental or COSMO-RS generated data. To estimate these binary interaction parameters, we minimize the squared error between calculate and measured pressures:

$$\min \sum_{i=1}^{n} (P_i - \widehat{P_i})^2$$

$$\text{s.t. Eqs. S1-S6.} \quad \text{(S7)}$$

Here $i = 1, \ldots, n$ is the index of the data points, P_i is the predicted pressure, and $\widehat{P}_i$ is the measured solubility at the same condition. After solving S7, the predicted $\widehat{P}_i$ are used to compute the average absolute deviation (AAD) as follows:

$$AAD = \frac{1}{n}\left(\sum_{i=1}^{n} |P_i - \widehat{P}_i|\right) \times 100\% \quad \text{(S8)}$$

In our work, the NRTL parameters are obtained using global optimization solver BARON(Sahinidis, 1996) with a specified time limit of 120 seconds. For process calculation, we only select those ILs for which AAD is below 10^{-2} with the estimated NRTL parameters.

S5

S6

S3. List of Feasible ILs

Table S1: Process performance of all ILs that meet the minimum required purity.

Cation	Anion	MW (g/mol)	H_{R32} (MPa)	H_{R125} (MPa)	Density (g/cm^3)	Viscosity (mPa.s)	Melting Point (K)	Eq Work (kJ/kg)
1-(3-methoxypropyl)-1-methylpyrrolidinium	tetrachloroferrate(iii)_hextuplet	355.92	2.77	22.57	1.356	26.56	267.95	124.14
1-(3-methoxypropyl)-1-methylpyrrolidinium	tetrachloroaluminate	327.06	2.81	22.30	1.240	27.97	265.52	124.41
1-(3-methoxypropyl)-1-methylpyrrolidinium	bcl4	310.89	2.90	22.51	1.232	35.72	251.28	125.41
ethyl-(3-methoxypropyl)-dimethylammonium	tetrachloroferrate(iii)_hextuplet	343.91	2.88	23.49	1.338	27.44	280.59	125.97
ethyl-(3-methoxypropyl)-dimethylammonium	tetrachloroaluminate	315.05	2.93	23.49	1.220	28.90	278.24	125.98
ethyl-(3-methoxypropyl)-dimethylammonium	tetrachlorogallate	357.80	2.86	22.14	1.402	28.90	280.36	126.78
ethyl-dimethyl-2-methoxyethylammonium	tetrachloroferrate(iii)_hextuplet	329.88	3.07	24.72	1.377	20.01	281.69	126.96
1-(2-methoxyethyl)-1-methylpyrrolidinium	tetrachloroaluminate	313.03	3.00	23.56	1.272	20.55	265.31	127.05
ethyl-dimethyl-propylammonium	chcl3	234.60	3.20	23.11	1.151	26.15	297.42	127.12
1-(2-methoxyethyl)-1-methylpyrrolidinium	tetrachloroferrate(iii)_hextuplet	341.89	2.96	23.53	1.396	19.52	267.85	127.24
ethyl-(3-methoxypropyl)-dimethylammonium	bcl4	298.88	3.04	24.03	1.210	36.85	264.45	127.38
ethyl-dimethylsulfonium	bf4	178.01	3.15	23.02	1.313	99.01	292.53	127.38
1-(2-methoxyethyl)-1-methylpyrrolidinium	tetrachlorogallate	355.78	2.93	22.19	1.463	20.56	267.60	127.46
1-(ethoxymethyl)-3-methyl-imidazolium	tetrachloroferrate(iii)_hextuplet	338.85	3.02	22.84	1.462	26.65	263.20	127.74
triethylsulfonium	chcl3	237.62	3.19	22.65	1.212	22.39	367.37	127.82
1-(2-methoxyethyl)-3-methylimidazolium	tetrachloroferrate(iii)_hextuplet	338.85	3.01	22.73	1.463	18.42	263.38	127.88
1-(3-methoxypropyl)-1-methylpyrrolidinium	tetrachlorogallate	369.81	2.74	21.15	1.418	27.97	267.71	128.03
ethyl-dimethyl-2-methoxyethylammonium	chcl3	250.60	3.21	24.13	1.177	30.65	286.09	128.04

S7

1-(2-methoxyethyl)-1-methylpyrrolidinium	bcl4	296.86	3.13	24.61	1.265	26.15	250.39	128.05
1-(ethoxymethyl)pyridinium	chcl3	256.56	3.26	23.62	1.273	33.21	255.82	128.13
1-(2-methoxyethyl)-1-methylpyrrolidinium	chcl3	262.61	3.00	21.07	1.204	30.02	269.46	128.25
4-ethyl-4-methylmorpholinium	bf4	217.02	3.15	23.45	1.269	231.66	246.22	128.39
n,n,n',n'-tetramethyl-1,2-ethanediamine	bcl4	269.84	3.16	22.53	1.234	23.16	272.53	128.50
diethyl-methyl-(2-methoxyethyl)ammonium	bcl4	298.88	3.05	21.96	1.217	26.69	261.58	128.51
tetramethylammonium	trifluoromethyltrifluoroborate	210.96	3.20	21.59	1.354	82.62	398.44	128.55
ethyl-dimethyl-propylammonium	tetrachloroferrate(iii)_hextuplet	313.88	3.08	22.24	1.363	17.21	290.60	128.66
1,1-dimethyl-pyrrolidinium	bclf3	203.44	3.03	21.30	1.270	17.00	170.86	128.68
di-ethyl-di-isopropylammonium	chcl3	248.62	3.18	22.23	1.139	24.75	381.46	128.74
1-ethyl-2,3-dimethyl-imidazolium	chcl3	243.56	3.07	21.23	1.245	19.03	294.66	128.85
tetramethylammonium	cyclopentadiene	139.24	3.22	22.71	0.926	270.58	876.86	129.01
1-propylpyridinium	chcl3	240.56	3.22	22.56	1.253	23.00	264.96	129.13
4543-96-8	bcl4	269.84	2.94	20.25	1.238	18.93	256.44	129.22
diethyl-methyl-(2-methoxyethyl)ammonium	tetrachloroferrate(iii)_hextuplet	343.91	2.89	21.07	1.345	19.88	277.71	129.23
ethyl-dimethyl-propylammonium	tetrachloroaluminate	285.02	3.15	22.66	1.232	18.10	288.02	129.39
n,n,n',n'-tetramethyl-1,2-ethanediamine	tetrachloroaluminate	286.01	3.05	21.30	1.243	18.28	287.59	129.43
1-(2-methoxyethyl)-3-methylimidazolium	tetrachlorogallate	352.74	3.00	21.73	1.534	19.39	263.13	129.53
butyltrimethylammonium	bcl4	268.85	3.23	22.94	1.214	28.00	276.27	129.56
1-(3-methoxypropyl)pyridinium	tetrachloroaluminate	321.01	2.92	20.86	1.302	26.36	252.33	129.65
1-ethyl-3-methyl-imidazolium	tetrachloroferrate(iii)_hextuplet	308.83	3.21	24.56	1.506	12.64	271.95	129.70
triethylsulfonium	tetrachloroferrate(iii)_hextuplet	316.91	3.16	22.88	1.423	14.81	342.42	129.73
tetramethylammonium	methyl-9h-fluorene-9-carboxylate	297.39	2.92	20.38	1.082	764.37	384.74	129.74
1-(3-methoxypropyl)pyridinium	bcl4	304.84	3.07	22.08	1.296	33.55	238.35	129.76
1-(2-methoxyethyl)pyridinium	tetrachloroaluminate	306.98	3.20	24.50	1.344	18.85	252.26	129.76
n,n-diethyl-n-methylethylammonium	tetrachloroaluminate	285.02	3.27	24.64	1.241	15.56	285.14	129.79
1-(3-methoxypropyl)-1-methylpiperidinium	tetrachloroaluminate	341.08	2.71	20.20	1.229	29.44	270.32	129.82

S8

1-(2-methoxyethyl)-3-methylimidazolium	tetrachloroaluminate	309.99	3.08	23.15	1.333	19.37	260.86	129.83
1-propylpyridinium	tetrachloroaluminate	290.98	3.19	22.71	1.324	16.04	259.58	129.85
1-ethyl-3-methylpyridinium	bcl4	274.81	3.27	23.05	1.320	16.85	260.48	129.88
1-(ethoxymethyl)-3-methyl-imidazolium	tetrachlorogallate	352.74	3.02	21.95	1.533	28.06	262.96	129.89
triethylphosphine	bcl4	271.79	3.03	21.28	1.246	23.13	272.92	129.94
ethyl-dimethyl-2-methoxyethylammonium	tetrachlorogallate	343.77	3.05	23.49	1.446	21.08	281.44	129.99
1-(3-methoxypropyl)pyridinium	tetrachloroferrate(iii)_hextuplet	349.87	2.87	20.69	1.425	25.05	254.71	130.00
4-(2-methoxyethyl)-4-methylmorpholinium	bclf3	263.50	3.23	23.27	1.262	53.81	178.10	130.00
1-(ethoxymethyl)-3-methyl-imidazolium	tetrachloroaluminate	309.99	3.10	23.38	1.332	28.04	260.69	130.00
1-acryloyloxypropyl-3-methyl-imidazolium	bcl4	347.86	2.92	21.07	1.281	129.97	257.90	130.10
1-(2-methoxyethyl)pyridinium	tetrachlorogallate	349.73	3.12	22.94	1.549	18.87	254.52	130.11
1-propenyl-3-methyl-imidazolium	tetrachloroferrate(iii)_hextuplet	320.83	3.15	23.03	1.468	16.85	277.61	130.13
ethyl-(3-methoxypropyl)-dimethylammonium	chcl3	264.62	2.95	20.21	1.148	42.51	283.96	130.25
n,n-diethyl-n-methylethylammonium	tetrachloroferrate(iii)_hextuplet	313.88	3.19	24.10	1.374	14.80	287.72	130.35
n,n-diethyl-n-methylethylammonium	tetrachlorogallate	327.77	3.17	22.94	1.446	15.58	287.47	130.37
triethylphosphine	tetrachloroaluminate	287.96	2.94	20.34	1.255	18.26	288.09	130.37
1-(2-ethoxy-2-oxoethyl)-3-methyl-imidazolium	tetrachloroaluminate	338.00	3.15	24.53	1.344	45.15	269.05	130.37
1-(2-methoxyethyl)-1-methylpiperidinium	tetrachloroaluminate	327.06	2.88	20.56	1.258	21.92	270.75	130.42
(2-ethoxyethyl)-ethyl-dimethylammonium	bcl4	298.88	2.96	20.68	1.214	31.24	263.87	130.43
(ethoxymethyl)-dimethyl-ethylammonium	bcl4	284.85	3.14	22.35	1.238	29.35	265.95	130.46
1-ethyl-2,3-dimethyl-imidazolium	tetrachloroaluminate	293.98	3.10	21.77	1.316	13.23	283.99	130.47
trifluoroethylamine	tetrachloroferrate(iii)_hextuplet	297.72	3.11	24.67	1.878	210.71	232.96	130.50
(2-ethoxyethyl)-ethyl-dimethylammonium	tetrachloroferrate(iii)_hextuplet	343.91	2.80	19.84	1.342	23.26	280.02	130.54
1-(2-methoxyethyl)pyridinium	tetrachloroferrate(iii)_hextuplet	335.85	3.13	23.97	1.476	17.92	254.76	130.55
1-ethyl-2,3-dimethyl-imidazolium	tetrachloroferrate(iii)_hextuplet	322.85	3.04	21.35	1.452	12.58	286.81	130.60

S9

1-acryloyloxypropyl-3-methyl-imidazolium	tetrachloroferrate(iii)_hextuplet	392.90	2.72	19.74	1.394	96.17	273.74	130.62
1-ethyl-1-methyl-pyrrolidinium	tetrachlorogallate	325.76	3.22	24.66	1.505	14.78	277.69	130.64
o-methyl-n,n,n,n-tetramethylisouronium	bcl4	283.82	2.87	19.55	1.290	16.79	266.50	130.65
tetra-ethylammonium	chcl3	248.62	3.05	20.63	1.137	23.58	325.24	130.68
(ethoxymethyl)-dimethyl-ethylammonium	tetrachloroferrate(iii)_hextuplet	329.88	2.95	20.70	1.374	21.95	282.85	130.73
1-(ethoxymethyl)pyridinium	tetrachloroaluminate	306.98	3.19	23.83	1.339	22.97	252.46	130.75
1-ethyl-2,3-dimethyl-imidazolium	bcl4	277.82	3.23	23.34	1.310	16.74	267.47	130.80
1-(2-ethoxy-2-oxoethyl)-3-methyl-imidazolium	tetrachloroferrate(iii)_hextuplet	366.86	3.07	23.86	1.464	42.89	271.53	130.84
1-propylpyridinium	tetrachloroferrate(iii)_hextuplet	319.85	3.12	22.18	1.462	15.26	262.21	130.95
acetylcholine_acetate	bf4	233.02	3.13	22.69	1.225	616.75	277.29	130.96
butyltrimethylammonium	chcl3	234.60	3.18	22.10	1.144	31.93	301.39	130.96
trifluoroethyldimethylamine	tetrachloroferrate(iii)_hextuplet	325.78	3.12	23.26	1.683	56.97	268.11	131.00
tetramethylammonium	9h-fluorene-9-carbonitrile	264.37	3.02	20.32	1.040	449.68	475.49	131.01
trifluoroethylmethylamine	tetrachloroferrate(iii)_hextuplet	311.75	3.20	25.08	1.766	102.64	252.70	131.01
(2-ethoxyethyl)-ethyl-dimethylammonium	tetrachloroaluminate	315.05	2.85	19.95	1.224	24.49	277.67	131.09
di-ethyl-di-isopropylammonium	tetrachloroferrate(iii)_hextuplet	327.91	3.10	21.93	1.340	16.11	357.50	131.21
triethylsulfonium	tetrachloroaluminate	288.04	3.22	23.28	1.287	15.58	339.41	131.23
n,n,n',n'-tetramethyl-1,2-ethanediamine	tetrachloroferrate(iii)_hextuplet	314.87	3.00	20.95	1.376	17.38	290.15	131.25
1-methyl-1-propylpyrrolidinium	tetrachloroaluminate	297.03	2.97	20.27	1.251	17.91	275.06	131.32
1-methyl-2-propylpyrazolium	tetrachloroaluminate	293.99	3.26	23.06	1.314	17.31	267.52	131.33
(ethoxymethyl)-dimethyl-ethylammonium	tetrachloroaluminate	301.02	3.01	21.05	1.247	23.10	280.39	131.34
1-(ethoxymethyl)pyridinium	tetrachloroferrate(iii)_hextuplet	335.85	3.10	23.20	1.471	21.84	254.96	131.36
1-propenyl-3-methyl-imidazolium	tetrachloroaluminate	291.97	3.23	23.75	1.330	17.72	274.89	131.37
propyl-dimethyl-isopropylammonium	chcl3	248.62	3.02	19.83	1.130	27.63	311.66	131.42
1-(3-cyanopropyl)-3-methylimidazolium	pf6	295.16	3.20	22.01	1.409	1608.84	312.43	131.42
1-ethyl-3-methyl-imidazolium	tetrachlorogallate	322.72	3.20	23.53	1.587	13.31	271.68	131.45
trifluoroethyldimethylamine	tetrachloroaluminate	296.91	3.27	25.08	1.529	59.83	265.41	131.52
tetramethylammonium	2,3,4,6-tetrachlorophenol	305.03	3.20	22.00	1.367	285.34	476.75	131.53

S10

butyltrimethylammonium	tetrachloroaluminate	285.02	3.10	21.33	1.225	22.09	291.46	131.55
(ethoxycarbonylmethyl)-ethyl-dimethylammonium	tetrachloroferrate(iii)_hextuplet	357.89	3.06	22.00	1.395	33.06	288.36	131.61
1,3-methyl-imidazolium	pf6	242.10	3.22	21.35	1.574	111.69	439.84	131.65
tetramethylammonium	2,4,6-trichlorophenol	270.59	3.22	21.46	1.291	320.77	552.97	131.65
(ethoxycarbonylmethyl)-ethyl-dimethylammonium	tetrachloroaluminate	329.03	3.13	22.47	1.277	34.82	285.94	131.66
1-(2-methoxy-2-oxoethyl)-3-methyl-imidazolium	pf6	300.14	3.22	22.13	1.504	408.03	328.40	131.75
1-propenyl-3-methyl-imidazolium	tetrachlorogallate	334.72	3.14	22.20	1.543	17.74	277.34	131.75
1-(ethoxymethyl)pyridinium	tetrachlorogallate	349.73	3.10	22.26	1.543	23.00	254.72	131.82
1-methyl-3-(1-methylethyl)imidazolium	bcl4	277.82	3.11	21.35	1.303	19.63	269.72	131.84
1-methyl-2-propylpyrazolium	tetrachloroferrate(iii)_hextuplet	322.85	3.18	22.43	1.449	16.46	270.17	131.86
1-(ethoxymethyl)-1-methylpyrrolidinium	bcl4	296.86	2.99	20.22	1.269	27.72	251.91	131.88
(ethoxycarbonylmethyl)-ethyl-dimethylammonium	chcl3	278.61	3.20	21.78	1.213	51.26	293.18	132.04
1-(3-methoxypropyl)-1-methylpyrrolidinium	tetrachloroindium	414.89	2.82	20.74	1.581	28.37	274.58	132.05
1-ethyl-2-methylpyrrolidine	tetrachlorogallate	325.76	3.21	22.37	1.494	19.11	281.47	132.05
1,3-diethylimidazolium	bcl4	277.82	3.06	20.48	1.308	19.20	283.26	132.12
1-(3-methoxypropyl)-1-methylpiperidinium	tetrachloroferrate(iii)_hextuplet	369.95	2.68	20.48	1.339	27.93	272.76	132.14
tetra-ethylammonium	tetrachloroaluminate	299.05	3.02	20.41	1.214	16.15	310.94	132.14
1-(2-ethoxyethyl)-1-methylpyrrolidinium	bcl4	310.89	2.83	19.49	1.232	30.65	250.68	132.24
1-ethyl-2-methylpyrrolidine	tetrachloroferrate(iii)_hextuplet	311.87	3.27	23.75	1.419	18.15	281.75	132.28
tetra-ethylammonium	tetrachloroferrate(iii)_hextuplet	327.91	2.98	20.32	1.338	15.35	313.66	132.47
4-cyano-1-butylpyridinium	tetrachloroferrate(iii)_hextuplet	358.88	3.14	22.12	1.396	102.84	273.14	132.55
n-allylpiperidine	tetrachloroferrate(iii)_hextuplet	323.88	3.23	22.30	1.410	24.30	277.25	132.55
diethyl-methyl-(2-methoxyethyl)ammonium	tetrachlorogallate	357.80	2.86	19.90	1.409	20.94	277.48	132.61
1-ethyl-2-methylpyrazolium	bclf3	214.43	3.25	21.34	1.319	17.43	175.86	132.63
ethyl-(3-methoxypropyl)-dimethylammonium	tetrachloroindium	402.88	2.93	21.74	1.568	29.33	287.02	132.65
1-methyl-1-propylpyrrolidinium	tetrachloroferrate(iii)_hextuplet	325.89	2.93	20.09	1.379	17.02	277.74	132.87

S11

diethyl-methyl-propylammonium	bcl4	282.88	3.05	20.06	1.191	23.86	270.05	132.87
4-cyano-1-butylpyridinium	tetrachloroaluminate	330.02	3.23	22.72	1.278	108.30	270.54	132.87
1-(2-methoxyethyl)-1-methylpyrrolidinium	tetrachloroindium	400.87	3.01	21.88	1.636	20.89	274.78	132.87
1-(2-ethoxy-2-oxoethyl)-3-methyl-imidazolium	tetrachlorogallate	380.75	3.07	22.98	1.529	45.16	271.29	132.94
4-(2-methoxyethyl)-4-methylmorpholinium	asf6	349.15	3.20	22.35	1.597	47.44	168.92	132.94
1-ethyl-pyridinium	bclf3	211.42	3.10	19.74	1.325	16.16	169.72	132.94
1-(3-methoxypropyl)pyridinium	tetrachlorogallate	363.76	2.86	19.68	1.492	26.37	254.49	132.95
4-(2-methoxyethyl)-4-methylmorpholinium	pf6	305.20	3.11	21.03	1.407	431.37	302.72	132.95
1-(3-methoxypropyl)-1-methylpiperidinium	bcl4	324.91	2.79	20.16	1.221	37.70	255.95	132.97
1-methyl-3-(1-methylethyl)imidazolium	tetrachloroaluminate	293.99	2.99	20.07	1.309	15.51	286.26	132.98
tetramethylammonium	bis(trifluoromethyl)imide	354.30	3.20	23.36	1.404	82.64	314.50	132.99
1-methylpyrrole	bis(trifluoromethyl)imide	362.27	3.10	21.30	1.508	15.26	156.00	133.03
trifluoroethyldimethylamine	tetrachlorogallate	339.66	3.18	23.27	1.768	59.98	267.83	133.07
1-ethyl-3,4-dimethylimidazolium	bcl4	277.82	3.05	20.39	1.307	17.44	269.92	133.09
1-ethyl-3-methylpyridinium	chcl3	240.56	3.05	20.14	1.254	19.11	286.52	133.14
1-(2-ethoxy-2-oxoethyl)-1-methylpyrrolidinium	tetrachloroaluminate	341.04	3.09	21.81	1.292	35.22	273.43	133.15
di-ethyl-di-isopropylammonium	tetrachlorogallate	341.80	3.07	20.77	1.407	16.97	357.20	133.34
1-methyl-3-(1-methylethyl)imidazolium	tetrachloroferrate(iii)_hextuplet	322.85	2.93	19.70	1.444	14.75	289.08	133.36
1,2-diethyl-4-fluoropyrazolium	bcl4	295.81	3.24	21.64	1.363	29.22	281.04	133.41
propyl-dimethyl-isopropylammonium	tetrachloroferrate(iii)_hextuplet	327.91	2.95	19.66	1.331	17.97	303.04	133.50
tetramethylammonium	2-hydroxybutanedioate	207.23	3.18	21.19	1.182	234.53	307.40	133.54
1-(2-methoxyethyl)pyridinium	tetrachloroindium	394.82	3.22	23.05	1.733	19.20	261.61	133.56
4-ethyl-4-methylmorpholinium	fluoradene	369.50	3.12	21.81	1.082	263.11	263.74	133.57
1-methyl-3-vinylimidazolium	bf4	195.96	3.17	19.60	1.336	109.92	261.65	133.58
1-methylpyrrole	9h-fluorene-9-carbonitrile	272.35	3.05	20.10	1.100	84.01	218.85	133.59
tetramethylammonium	2,4,5-trichlorophenol	270.59	3.14	19.86	1.291	282.53	439.89	133.60
butyltrimethylammonium	tetrachloroferrate(iii)_hextuplet	313.88	3.02	20.78	1.356	21.00	294.05	133.61

S12

di-ethyl-di-isopropylammonium	tetrachloroaluminate	299.05	3.15	22.08	1.216	16.96	354.38	133.68
1-(2-ethoxyethyl)pyridinium	bcl4	304.84	3.05	20.58	1.295	28.65	238.96	133.71
1-(2-ethoxy-2-oxoethyl)-1-methylpyrrolidinium	bcl4	324.87	3.25	23.20	1.287	45.00	258.75	133.72
4-(ethoxymethyl)-4-methylmorpholinium	bclf3	263.50	3.16	20.97	1.261	58.54	179.49	133.77
1,3-bis(3-cyanopropyl)imidazolium	pf6	348.23	3.13	21.21	1.311	11879.83	285.86	133.85
butyltrimethylammonium	tetrachlorogallate	327.77	3.02	20.00	1.427	22.11	293.79	133.90
1-ethyl-nicotinic-acid-ethylester	tetrachloroferrate(iii)_hextuplet	377.88	2.89	19.79	1.432	43.23	276.87	133.92
1-(2-methoxy-2-oxoethyl)-3-methyl-imidazolium	bf4	241.99	3.19	21.12	1.341	303.05	250.21	133.94
ethyl-dimethyl-2-methoxyethylammonium	tetrachloroindium	388.86	3.13	23.17	1.623	21.43	288.39	133.95
tetramethylammonium	2,3-dichlorophenol	236.14	3.23	20.24	1.218	391.14	394.79	133.97
1-propyl-3-methyl-imidazolium	bcl4	277.82	3.11	21.13	1.306	21.17	253.63	133.98
1-ethyl-2,3-dimethyl-imidazolium	tetrachlorogallate	336.74	3.01	20.37	1.526	13.25	286.53	134.02
n,n-diethyl-n-methylethylammonium	tetrachloroindium	372.86	3.23	22.53	1.632	15.86	294.78	134.03
1-ethyl-nicotinic-acid-ethylester	tetrachloroaluminate	349.02	2.94	19.97	1.318	45.53	274.38	134.03
tetramethylammonium	pentachlorophenol	339.48	3.19	22.01	1.439	253.14	636.71	134.12
n-propylpiperidine	bcl4	280.86	3.20	21.05	1.255	30.72	253.71	134.12
1-(ethoxymethyl)-3-methyl-imidazolium	tetrachloroindium	397.82	3.13	22.16	1.714	28.55	270.07	134.17
1-ethyl-nicotinic-acid-ethylester	bcl4	332.85	3.10	21.14	1.313	58.19	259.77	134.22
1-(2-ethoxy-2-oxoethyl)pyridinium	tetrachlorogallate	377.74	3.22	24.32	1.541	41.39	262.47	134.23
n-ethyl-n-methyl-n-(1-methylethyl)-2-propylammonium	bcl4	296.90	3.11	20.35	1.186	22.47	292.94	134.26
1-(2-methoxyethyl)-3-methylimidazolium	tetrachloroindium	397.82	3.11	21.91	1.715	19.73	270.25	134.37
(ethoxycarbonylmethyl)-ethyl-dimethylammonium	tetrachlorogallate	371.78	3.04	21.05	1.459	34.82	288.12	134.38
1,1-dimethyl-pyrrolidinium	bisoxalatoborate	287.03	3.26	22.18	1.336	21.97	228.41	134.41
n,n,n',n'-tetramethyl-1,3-propanediamine	carbazole	297.44	3.15	20.09	1.006	149.06	250.76	134.42
1-(2-ethoxy-2-oxoethyl)-1-methylpyrrolidinium	tetrachloroferrate(iii)_hextuplet	369.90	3.03	21.44	1.407	33.44	275.93	134.47
acetylcholine_acetate	sbf6	381.96	2.96	20.03	1.770	82.09	203.79	134.47

S13

1,3-bis(3-cyanopropyl)imidazolium	asf6	392.18	3.19	22.26	1.469	1298.96	179.21	134.53
1-methylpyrrole	fluoradene	321.42	3.19	21.45	1.107	115.76	231.05	134.58
1-tert-butyl-pyridinium	tetrachloroferrate(iii)_hextuplet	333.87	3.02	19.94	1.420	14.08	301.78	134.81
1-ethyl-3-methylpyridinium	tetrachlorogallate	333.73	3.04	20.10	1.539	13.35	279.43	134.84
1-tert-butyl-pyridinium	bcl4	288.84	3.19	21.37	1.285	18.81	281.65	134.86
1-propyl-3-methyl-imidazolium	tetrachloroaluminate	293.99	2.97	19.65	1.312	16.73	269.17	134.87
ethyl-(3-hydroxypropyl)-dimethylammonium	bf4	219.04	3.21	19.79	1.181	324.29	255.18	134.90
4-(2-ethoxyethyl)-4-methylmorpholinium	tetrachloroindium	430.89	3.19	24.28	1.610	46.75	272.08	134.92
(ethoxymethyl)-dimethyl-ethylammonium	tetrachlorogallate	343.77	2.93	19.78	1.442	23.11	282.61	134.93
1-ethyl-3-methyl-imidazolium	clo4	210.62	3.22	19.54	1.365	93.26	282.25	135.05
1-methyl-3-(1-methylethyl)imidazolium	chcl3	243.56	3.06	19.68	1.238	22.32	297.20	135.12
1-(2-ethoxy-2-oxoethyl)-1-methylpyrrolidinium	chcl3	290.62	3.13	20.61	1.232	52.06	278.33	135.13
1,1-dimethyl-pyrrolidinium	asf6	289.10	3.18	21.00	1.701	15.41	158.36	135.17
n-ethyl-n-methyl-n-(1-methylethyl)-2-propylammonium	tetrachloroaluminate	313.07	3.01	19.54	1.196	17.61	308.42	135.19
1-propylpyridinium	tetrachlorogallate	333.73	3.11	21.29	1.537	16.07	261.96	135.23
1-methylpyrrole	methyl-9h-fluorene-9-carboxylate	305.37	3.04	20.20	1.138	143.09	208.10	135.23
1-(ethoxymethyl)pyridinium	tetrachloroindium	394.82	3.20	22.42	1.727	23.41	261.82	135.42
n-ethyl-n-methyl-n-(1-methylethyl)-2-propylammonium	tetrachloroferrate(iii)_hextuplet	341.94	2.98	19.58	1.313	16.72	311.05	135.44
n-propylpiperidine	tetrachloroaluminate	297.03	3.13	20.33	1.264	24.22	269.38	135.61
4-ethyl-4-methylmorpholinium	cyclopentadiene	195.30	3.25	19.64	1.010	134.37	269.14	135.78
n,n-dimethylcyclohexylamine	tetrachloroferrate(iii)_hextuplet	325.89	3.27	21.50	1.399	28.98	296.72	135.88
1-(2-ethoxy-2-oxoethyl)-1-methylpyrrolidinium	tetrachlorogallate	383.79	3.01	20.45	1.470	35.22	275.68	136.03
1-methylindole	bf4	218.99	3.21	19.71	1.326	162.30	246.90	136.05
1-methyl-2-propylpyrazolium	tetrachlorogallate	336.74	3.17	21.55	1.523	17.34	269.91	136.09
1-methyl-3-(2,2,2-trifluoroethyl)imidazolium	bcl4	317.76	3.04	19.56	1.515	52.74	240.89	136.22
1,3-bis(3-cyanopropyl)imidazolium	bf4	290.08	3.04	19.67	1.176	9185.20	229.93	136.26

S14

ethyl-(3-hydroxypropyl)-dimethylammonium	sbf6	367.98	3.22	20.91	1.745	43.44	190.23	136.29
1-methyl-3-vinylimidazolium	pf6	254.11	3.20	19.85	1.534	152.76	362.75	136.34
diethyl-methyl-(2-methoxyethyl)ammonium	tetrachloroaluminate	315.05	2.93	21.12	1.226	20.93	275.36	136.36
ethyl-phenyl-methylsulfonium	tetrachloroaluminate	322.06	3.05	19.52	1.309	19.90	300.21	136.65
diethyl-methyl-(2-methoxyethyl)ammonium	tetrachloroindium	402.88	2.94	19.64	1.577	21.25	284.13	136.69
triethylsulfonium	tetrachlorogallate	330.80	3.12	21.70	1.496	15.60	342.17	136.76
1-(2-ethoxy-2-oxoethyl)-3-methyl-imidazolium	tetrachloroindium	425.83	3.19	23.31	1.697	45.86	278.30	136.78
4-(dimethylamino)-1-methylpyridinium	bcl4	289.83	3.21	20.79	1.321	14.57	271.26	136.90
3,7-dihydro-1,3,7,9-tetramethyl-2,6-dioxo-1h-purinium	sbf6	444.98	3.16	23.31	1.835	173.49	218.09	136.91
n-propylpiperidine	tetrachloroferrate(iii)_hextuplet	325.89	3.09	20.17	1.393	23.02	272.05	136.97
ethyl-phenyl-methylsulfonium	bcl4	305.89	3.22	21.20	1.303	25.33	283.87	137.13
1-methyl-3-(3-oxobutyl)-imidazolium	clo4	252.65	3.21	20.57	1.317	240.60	287.81	137.14
1-(phenylmethyl)pyridinium	chcl3	288.60	3.25	20.98	1.241	45.85	281.32	137.26
4-(ethoxymethyl)-4-methylmorpholinium	asf6	349.15	3.11	20.18	1.597	51.61	170.52	137.33
1-butyl-pyridinium	bcl4	288.84	3.08	19.48	1.273	26.10	245.47	137.37
1-(3-methoxypropyl)pyridinium	tetrachloroindium	408.85	2.96	19.76	1.664	26.80	261.28	137.40
n,n,n',n'-tetramethyl-1,2-ethanediamine	tetrachloroindium	373.84	3.05	19.81	1.634	18.63	297.25	137.43
1-(3-cyanopropyl)-3-methylimidazolium	bisoxalatoborate	337.05	3.25	21.89	1.313	242.12	232.63	137.57
1-(phenylmethyl)pyridinium	tetrachloroferrate(iii)_hextuplet	367.89	3.06	19.83	1.417	29.51	276.65	137.60
1-propenyl-3-methyl-imidazolium	tetrachloroindium	379.81	3.24	22.33	1.736	18.08	285.05	137.60
1-methyl-2-(phenylmethyl)pyrazolium	chcl3	291.61	3.22	20.58	1.232	49.23	287.32	137.89
1-propylpyridinium	tetrachloroindium	378.82	3.22	21.45	1.729	16.37	269.44	137.97
ethyl-dimethyl-propylammonium	tetrachlorogallate	327.77	3.06	21.22	1.435	18.12	290.35	137.99
1-methyl-1-propylpyrrolidinium	bcl4	280.86	3.10	21.62	1.242	22.74	259.33	138.01
1-[2-(butylamino)-2-oxoethyl]-3-methyl-imidazolium	bcl4	348.90	3.13	20.45	1.244	121.92	257.17	138.03
ethyl-dimethyl-propylammonium	tetrachloroindium	372.86	3.14	21.01	1.620	18.44	297.67	138.06
1,3-dimethoxyimidazolium	asf6	318.05	3.18	19.92	1.813	21.36	161.65	138.16

S15

1-ethyl-2,3-dimethyl-imidazolium	tetrachloroindium	381.82	3.09	20.24	1.715	13.49	294.53	138.23
1-methyl-2-(phenylmethyl)pyrazolium	tetrachloroferrate(iii)_hextuplet	370.89	3.04	19.55	1.407	31.61	282.35	138.44
1-methyl-2-(phenylmethyl)pyrazolium	tetrachloroaluminate	342.03	3.12	20.10	1.292	33.29	279.56	138.56
1-ethyl-2-methylpyrrolidine	tetrachloroindium	370.84	3.27	21.99	1.686	19.47	289.47	138.57
ethyl-dimethylsulfonium	2,6-dichlorophenol	253.19	3.21	19.57	1.249	96.96	283.33	138.57
4-cyano-1-butylpyridinium	tetrachlorogallate	372.77	3.16	21.41	1.460	108.30	272.88	138.63
1-(2-methoxyethyl)-1-methylpiperidinium	bcl4	310.89	2.98	21.15	1.250	27.97	255.71	138.68
n,n-dimethylcyclohexylamine	tetrachlorogallate	339.78	3.22	20.38	1.470	30.51	296.42	138.69
1,3-dihydro-1-methyl-5-phenyl-1,4-benzodiazepin-2-one	tetrachloroaluminate	420.10	3.21	21.59	1.305	82.06	313.16	138.71
1-methylpyrrole	trifluoromethyltrifluoroborate	218.94	3.24	21.97	1.461	15.24	173.59	138.78
di-ethyl-di-isopropylammonium	tetrachloroindium	386.88	3.13	20.39	1.582	17.25	366.03	138.95
1-ethyl-2-methylpyrazolium	asf6	300.08	3.23	19.61	1.739	15.76	166.94	139.01
3,7-dihydro-1,3,7,9-tetramethyl-2,6-dioxo-1h-purinium	fluoradene	448.52	3.25	23.90	1.157	1325.81	321.18	139.04
1-(cyanomethyl)pyridinium	bis(trifluoromethyl)imide	399.30	3.18	21.13	1.463	101.67	173.49	139.05
dimethylethylamine	fluoradene	313.44	3.26	20.32	1.059	172.62	263.77	139.13
cyanomethyl-dimethyl-ethylammonium	9h-fluorene-9-carbonitrile	303.40	3.20	20.31	1.043	473.69	266.14	139.22
(ethoxymethyl)-dimethyl-ethylammonium	tetrachloroindium	388.86	3.00	19.57	1.619	23.50	289.56	139.22
1,3-dihydro-1-methyl-5-phenyl-1,4-benzodiazepin-2-one	tetrachloroferrate(iii)_hextuplet	448.96	3.14	21.37	1.399	77.77	316.25	139.39
1-(3-cyanopropyl)-3-methylimidazolium	thiocyanate	208.29	3.26	21.33	1.087	267.50	274.58	139.44
1-(phenylmethyl)pyridinium	tetrachloroaluminate	339.03	3.14	20.45	1.301	31.08	273.88	139.58
1-benzyl-3-methyl-1,2,3-triazolium	tetrachloroaluminate	343.02	3.12	19.93	1.320	37.53	275.00	139.63
4,5-dichloro-1-ethyl-3-methylimidazolium	tetrachloroaluminate	348.85	3.19	20.43	1.476	23.21	317.18	139.68
n,n-diethylaniline	bcl4	302.87	3.16	19.50	1.234	39.12	272.71	139.73
1-methylindole	pf6	277.14	3.18	19.48	1.505	221.45	345.03	139.79
(ethoxycarbonylmethyl)-ethyl-dimethylammonium	tetrachloroindium	416.87	3.13	21.00	1.625	35.34	294.96	139.83
4-ethyl-4-methylmorpholinium	sbf6	365.96	3.09	20.84	1.859	31.52	173.75	139.87
1-ethyl-3-methylpyridinium	tetrachloroferrate(iii)_hextuplet	319.85	3.07	21.15	1.463	12.68	279.69	140.21

S16

1-butyl-imidazolium	tetrachloroferrate(iii)_hextuplet	322.85	3.19	19.70	1.456	29.31	254.24	140.22
1-methylindole	asf6	321.10	3.25	20.19	1.726	24.48	165.75	140.25
1,2-diethyl-4-fluoropyrazolium	chcl3	261.55	3.17	19.90	1.305	33.31	312.58	140.33
1-benzyl-3-methyl-imidazolium	bcl4	325.86	3.09	19.54	1.285	45.95	266.64	140.51
1-ethyl-2-methylpyrazolium	bisoxalatoborate	298.02	3.25	19.90	1.371	22.38	231.00	140.77
1-[2-(2-methoxyethoxy)ethyl]-3-methyl-imidazolium	i3	565.96	3.05	21.23	2.235	42.20	453.18	140.78
collidine	tetrachloroferrate(iii)_hextuplet	319.85	3.19	20.54	1.463	14.78	327.63	140.86
trimethylethylammonium	2,4,6-tribromophenol	417.96	3.26	20.52	1.834	73.61	351.47	140.88
diethylbenzylamine	bcl4	316.89	3.21	19.74	1.210	50.41	270.85	140.92
ethyl-tetrahydrothiophenium	bisoxalatoborate	304.08	3.23	19.77	1.373	22.49	244.45	140.93
butyltrimethylammonium	tetrachloroindium	372.86	3.09	19.88	1.612	22.50	301.12	141.11
4,5-dichloro-1-ethyl-3-methylimidazolium	tetrachloroferrate(iii)_hextuplet	377.71	3.13	20.09	1.602	22.06	320.08	141.21
1-(cyanomethyl)pyridinium	9h-fluorene-9-carbonitrile	309.37	3.24	20.56	1.112	522.33	237.06	141.35
1-benzyl-3-methyl-4-(ethyl-2-ol)-1,2,3-triazolium	tetrachloroaluminate	387.07	3.17	19.98	1.279	43.81	274.65	141.74
1,3-bis(3-cyanopropyl)imidazolium	bisoxalatoborate	390.12	3.13	20.26	1.245	1707.31	230.15	141.75
1-ethyl-2,6-dimethylpyridinium	tetrachloroindium	392.85	3.15	19.82	1.677	13.44	299.90	141.90
tetramethylammonium	fluoradene	313.44	3.06	21.20	1.056	617.28	497.05	141.91
1,3-dimethoxyimidazolium	bismalonatoborate	344.04	3.26	20.78	1.381	138.47	208.23	142.06
1-methyl-3-(2-oxo-2-propoxyethyl)-imidazolium	chcl3	301.60	3.15	19.59	1.252	80.67	272.34	142.22
1-(2-hydroxyethyl)-3-methylimidazolium	fluoradene	366.46	3.26	20.61	1.108	254.92	249.46	142.42
4-(ethoxymethyl)-4-methylmorpholinium	bisoxalatoborate	347.09	3.21	19.58	1.313	70.10	232.11	142.60
n-isopropyl-benzothiazolium	bcl4	330.90	3.24	19.61	1.346	24.39	278.79	142.75
ethyl-dimethyl-2-methoxyethylammonium	bis(fluorosulfonyl)imide	312.36	3.25	19.55	1.348	23.61	248.91	142.81
1,3-dihydro-1-methyl-5-phenyl-1,4-benzodiazepin-2-one	tetrachlorogallate	462.85	3.13	20.34	1.450	81.92	315.96	142.85
1-ethyl-2,6-dimethylpyridinium	tetrachloroferrate(iii)_hextuplet	333.87	3.11	21.16	1.426	12.54	292.10	143.44
1-ethyl-3-methylpyridinium	tetrachloroaluminate	290.98	3.12	21.38	1.326	13.33	276.89	143.69
1,3-dihydro-3-hydroxy-1-methyl-5-phenyl-1,4-benzodiazepin-2-one	tetrachloroaluminate	436.10	3.22	20.36	1.334	68.58	316.01	144.01

S17

1,3-dihydro-3-hydroxy-1-methyl-5-phenyl-1,4-benzodiazepin-2-one	tetrachloroferrate(iii)_hextuplet	464.96	3.16	20.13	1.426	64.99	319.11	144.41
triethylsulfonium	tetrachloroindium	375.88	3.19	21.37	1.686	15.89	350.81	144.77
tetra-ethylammonium	bcl4	282.88	3.13	21.47	1.203	20.55	294.96	145.65
1-(2-methoxyethyl)-1-methylpiperidinium	tetrachloroferrate(iii)_hextuplet	355.92	2.84	20.56	1.375	20.81	273.32	145.68
acetylcholine_acetate	2,4,6-tribromophenol	476.00	3.24	20.20	1.729	406.25	345.08	146.66
1-ethyl-2,6-dimethylpyridinium	tetrachloroaluminate	305.01	3.17	21.52	1.298	13.20	289.28	146.70
1,3-dihydro-1-methyl-5-phenyl-1,4-benzodiazepin-2-one	tetrachloroindium	507.94	3.23	20.30	1.583	82.69	324.75	146.92
1-(2-ethoxyethyl)-1-methylpiperidinium	i3	553.00	3.24	21.50	2.199	21.55	520.14	149.38
ethyl-(3-hydroxypropyl)-dimethylammonium	fluoradene	371.52	3.15	20.80	1.042	353.09	269.55	150.64
1-methyl-3-(3-oxobutyl)-imidazolium	bis(fluorosulfonyl)imide	333.33	3.27	21.27	1.419	36.15	246.79	151.46
3-(cyanomethyl)-1-methyl-imidazolium	fluoradene	361.44	3.21	20.93	1.112	749.29	259.40	152.96
1-[2-(diethylamino)-2-oxoethyl]-3-methyl-imidazolium	bcl4	348.90	2.92	20.42	1.245	84.86	267.73	153.07
n-allylpiperidine	tetrachlorogallate	337.77	3.19	21.15	1.482	25.59	276.97	154.50
1-acryloyloxypropyl-3-methyl-imidazolium	tetrachloroaluminate	364.03	2.77	19.93	1.287	101.35	271.44	154.79
1-ethyl-2,6-dimethylpyridinium	chcl3	254.59	3.16	21.37	1.228	19.14	301.33	157.62
n-allylpiperidine	tetrachloroindium	382.85	3.27	21.11	1.666	26.04	284.76	166.42
1-methyl-3-(2-oxo-2-propoxyethyl)-imidazolium	bcl4	335.85	3.07	20.75	1.304	69.58	254.52	170.62
propyl-dimethyl-isopropylammonium	bcl4	282.88	3.11	20.92	1.197	24.06	285.09	176.29
59009-70-0	i3	702.15	3.08	19.82	1.820	233.35	595.81	177.56
triethylphosphine	tetrachloroferrate(iii)_hextuplet	316.82	2.88	19.99	1.388	17.36	290.68	193.41
1-[2-(butylmethylamino)-2-oxoethyl]-3-methyl-imidazolium	i3	591.01	3.12	20.14	2.077	77.27	506.92	200.86
1-(2-ethoxy-2-oxoethyl)-1-methylpyrrolidinium	tetrachloroindium	428.88	3.09	20.32	1.631	35.72	282.75	201.15
acetylcholine_acetate	fluoradene	385.50	2.90	19.78	1.067	659.98	287.33	203.27
3-(3-carboxypropyl)-1-methyl-imidazolium	fluoradene	408.49	3.18	20.18	1.107	687.88	263.20	211.24
(ethoxymethyl)-dimethyl-ethylammonium	chcl3	250.60	3.04	20.58	1.174	33.62	287.40	213.39

S18

S19

S4. Process Performance Evaluation of Existing ILs

We select four experimentally well-characterized ILs known for their favorable solubility properties: 1-ethyl-3-methylimidazolium thiocyanate ([EMIM][SCN]), 1-ethyl-3-methylimidazolium dicyanamide ([EMIM][DCA]), 1-ethyl-3-methylimidazolium trifluoromethanesulfonate ([EMIM][OTF]), and 1-butyl-3-methylimidazolium dicyanamide ([BMIM][DCA]). The optimized performance results of these ILs, including equivalent energy consumption and CO_2-equivalent emissions for separating one kilogram of R-410A refrigerant, are presented in Table S2. We observe that, each of these ILs have low H_{R32} signifying high R-32 absorption. Among the ILs evaluated, [EMIM][SCN] demonstrates the superior performance, requiring the least energy (143.16 kJ/kg) and the lowest emissions (0.016 kg CO_2-eq/kg).

Table S2. Solubility and optimized process performance for four well-known ILs. Henry's constants are computed from COSMO-RS at 298.15 K, and corresponding process performance and emission are obtained from SPICE.

ILs	H_{R32} (MPa)	H_{R125} (MPa)	Eq. Work (kJ/kg)	CO_2-eqv emission (kg/kg)
[EMIM][SCN]	2.63	12.44	143.16	0.016
[EMIM][DCA]	2.46	9.86	154.60	0.017
[EMIM][OTF]	2.36	8.21	169.07	0.018
[BMIM][DCA]	2.19	6.72	193.95	0.021

S20

S5. Sigma Profiles and Excess Gibbs Free Energy Analysis

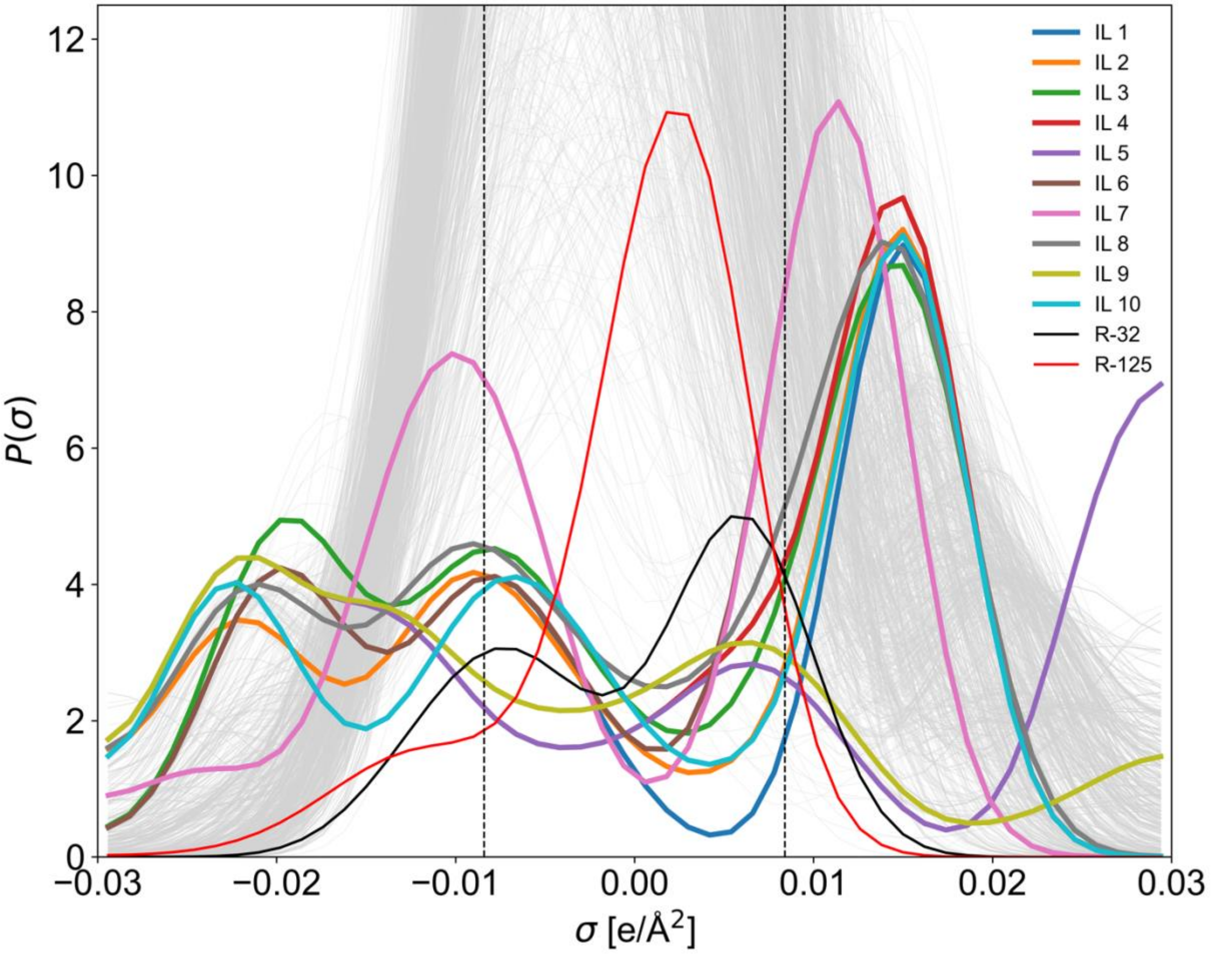

Figure S3: Sigma profiles of the top 10 ILs based on the highest R-32 selectivity. The sigma profiles are not exhibiting any regular behavior.

The ILs with the highest R-32 selectivity as shown in Figure S3 show that these ILs minimize the Van Der Waals (VdW) repulsions with R-125, troughing near zero. They also exhibit strong H-bond donor peaks overlapping the H-bond donor peaks of R-32, suggesting high VDW/electrostatic repulsion with R-32. The ILs also exhibit extremely strong H-bond donor peaks, which would result in high R-125 electrostatic repulsions. Therefore, while these ILs have high selectivity, the absorption of either R-32 or R-125 is extremely low.

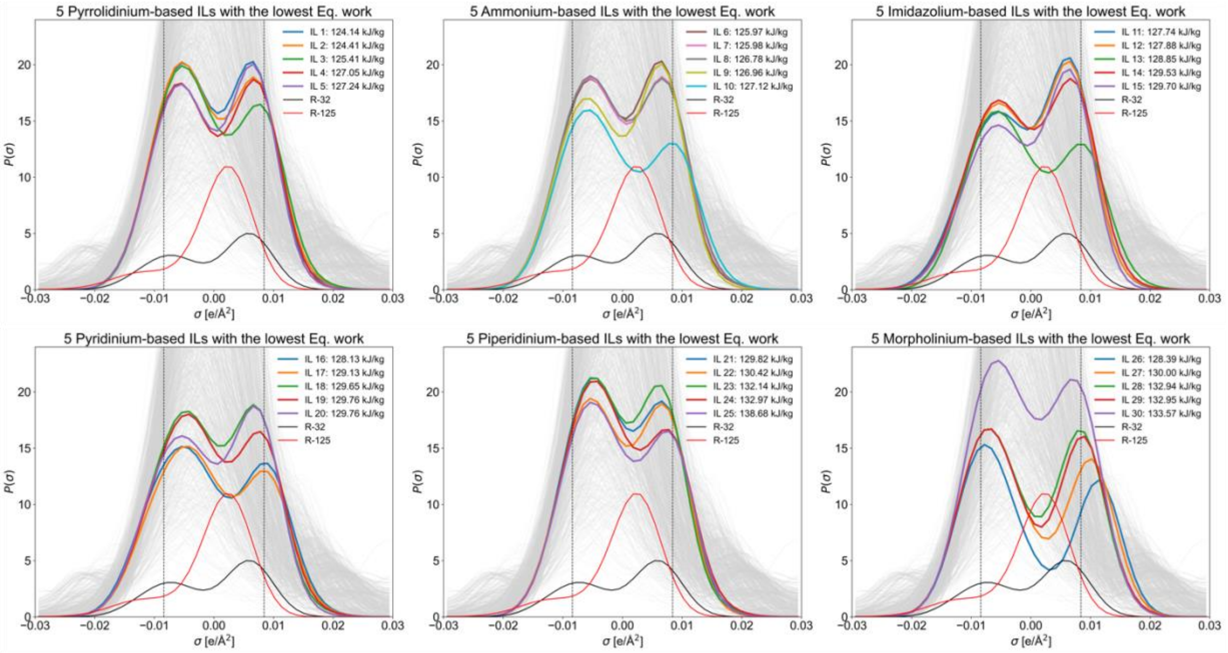

Figure S4: Sigma profiles of the top 5 feasible ILs based on process performance from each cationic family.

S6. Analysis of Pearson Correlation of Sigma Profiles of Process Feasible and Infeasible ILs

We analyze the sigma profiles of feasible ILs (meeting the minimum required purity) and infeasible ILs (not meeting the minimum required purity). For this, we select three feasible ILs (see Figure S5) and observe that as the peaks of the ILs in the neutral zone shift to the right, the required process energy becomes higher. In Figure S5(b), we show the sigma profiles of a feasible IL with two others infeasible ILs. This leads to an interesting observation. While it may be possible to discard ILs based on very low Pearson correlations, it may still be possible to have multiple ILs with similar trends in sigma profiles, with some ILs meeting the minimum required purity and others being infeasible. Therefore, although Pearson correlation may serve as a *material-centric screening* tool in addition to the solubility parameters, the final selection of IL candidates may be achieved only after *process optimization*. This again underscores the need for a *combined multi-scale* approach for the discovery of ILs for separation application.

(a)

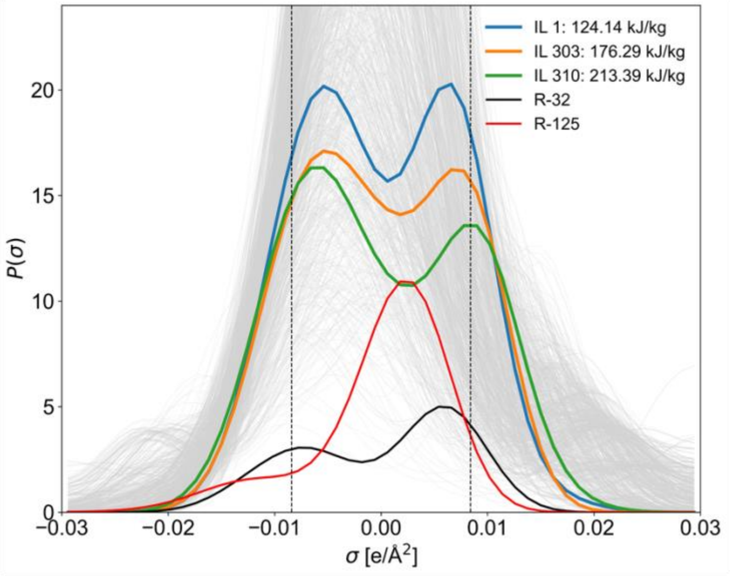

	IL 1	IL 303	IL 310
r_{R32}	0.95	0.94	0.89
r_{R125}	0.77	0.75	0.64
γ_{R32}^{∞}	1.64	1.84	1.80
γ_{R125}^{∞}	16.40	15.20	14.95

(b)

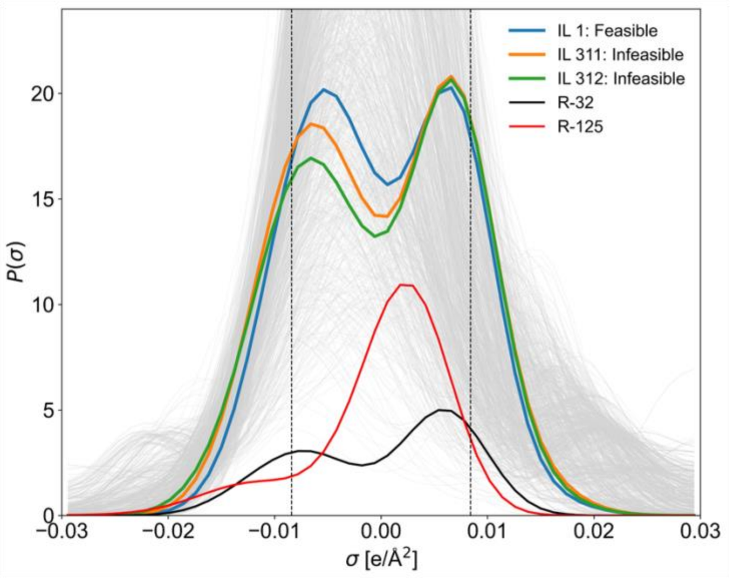

	IL 1	IL 311	IL 312
r_{R32}	0.95	0.97	0.98
r_{R125}	0.77	0.74	0.75
γ_{R32}^{∞}	1.64	1.85	1.93
γ_{R125}^{∞}	16.40	19.86	18.26

S23

Figure S5. (a) Sigma profiles comparison among feasible ILs. (b) Sigma profiles comparison among feasible and two infeasible ILs. IL1:1-(3-methoxypropyl)-1-methylpyrrolidinium tetrachloroferrate, IL303: propyl-dimethyl-isopropylammonium bcl4, IL310: (ethoxymethyl)dimethyl-ethylammonium chcl3, IL311: 4-(3-methoxypropyl)-4-methylmorpholinium tetrachloroferrate, IL312: 1-methyl-3-(3-oxobutyl)-imidazolium tetrachloroferrate.

Table S3. Abbreviations and full names of the top 10 feasible ILs based on process performance.

ILs	Abbreviation	Full Name
IL 1	[MOPMPy][FeCl4]	1-(3-methoxypropyl)-1-methylpyrrolidinium tetrachloroferrate(III)
IL2	[MOPMPy][AlCl4]	1-(3-methoxypropyl)-1-methylpyrrolidinium tetrachloroaluminate
IL3	[MOPMPy][BCl4]	1-(3-methoxypropyl)-1-methylpyrrolidinium tetrachloroborate
IL 4	[E-MOPAm][FeCl4]	ethyl-(3-methoxypropyl)-dimethylammonium tetrachloroferrate(III)
IL 5	[E-MOPAm][AlCl4]	ethyl-(3-methoxypropyl)-dimethylammonium tetrachloroaluminate
IL 6	[E-MOPAm][GaCl4]	ethyl-(3-methoxypropyl)-dimethylammonium tetrachlorogallate
IL 7	[E-MEAm][FeCl4]	ethyl-dimethyl-2-methoxyethylammonium tetrachloroferrate(III)
IL 8	[M-OPMPy][AlCl4]	1-(2-methoxyethyl)-1-methylpyrrolidinium tetrachloroaluminate
IL 9	[E-MPAm][CHCl3]	ethyl-dimethyl-propylammonium chloroformate
IL 10	[M-OPMPy][FeCl4]	1-(2-methoxyethyl)-1-methylpyrrolidinium tetrachloroferrate(III)

S7. Comparison of COSMO predicted Henry's constants with experimental data

Table S4: List of Experimental and COSMO-calculated Henry's constants.

IL Name	T (K)	Source	H_{R32}^{exp} (MPa)	H_{R32}^{cal} (MPa)	H_{R125}^{exp} (MPa)	H_{R125}^{cal} (MPa)
1-ethyl-3-methylimidazolium Thiocyanate	283.15	(Asensio-Delgado et al., 2021a, 2021b)	2.61	2.33	12.9	7.71
1-ethyl-3-methylimidazolium Thiocyanate	293.15	(Asensio-Delgado et al., 2021a, 2021b)	3.16	2.94	17.6	9.41
1-ethyl-3-methylimidazolium Thiocyanate	303.15	(Asensio-Delgado et al., 2021a, 2021b)	4.08	3.65	20.6	11.26
1-ethyl-3-methylimidazolium Thiocyanate	313.15	(Asensio-Delgado et al., 2021a, 2021b)	4.86	4.44	28.4	13.24
1-butyl-3-methylimidazolium Tetrafluoroborate	298.15	(Morais et al., 2020)	1.54	2.58	4.19	5.88
1-butyl-3-methylimidazolium Hexafluorophosphate	298.15	(Morais et al., 2020)	1.24	2.79	4.34	7.37
1-ethyl-3-methylimidazolium Trifluoromethansulfonate	303.15	(Sosa et al., 2019)	1.61	3.26	3.35	7.42
1-hexyl-3-methylimidazolium Trifluoromethansulfonate	303.15	(He et al., 2017)	1.59	2.66	2.43	4.03
1-hexyl-3-methylimidazolium Chloride	298.15	(Baca et al., 2021)	2	2.24	1.16	1.89
1-hexyl-3-methylimidazolium Bromine	298.15	(Baca et al., 2021)	1.71	2.38	2.56	2.46
1-ethyl-3-methylimidazolium Bis(trifluoromethylsulfonyl)imide	298.15	(Shiflett et al., 2006; Shiflett and Yokozeki, 2008)	1.09	2.47	1.76	4.76

S24

1-ethyl-3-methylimidazolium Acetate	303.15	(Sosa et al., 2019)	1.53	3.14	1.85	3.79
1-ethyl-3-methylimidazolium Dicyanamide	283.15	(Asensio-Delgado et al., 2021b)	1.81	2.20	8.27	6.17
1-ethyl-3-methylimidazolium Dicyanamide	293.15	(Asensio-Delgado et al., 2021b)	2.41	2.76	11.14	7.47
1-ethyl-3-methylimidazolium Dicyanamide	303.15	(Asensio-Delgado et al., 2021b)	2.93	3.40	14.55	8.89
1-ethyl-3-methylimidazolium Dicyanamide	313.15	(Asensio-Delgado et al., 2021b)	3.66	4.13	18.86	10.41
1-ethyl-3-methylimidazolium Dicyanamide	323.15	(Asensio-Delgado et al., 2021b)	5.03	4.94	21.85	12.02
1-butyl-3-methylimidazolium Acetate	298.15	(Morais et al., 2020)	1.2	2.81	0.81	2.93
1-butyl-3-methylimidazolium Dicyanamide	283.15	(Asensio-Delgado et al., 2021b)	1.51	1.96	4.83	4.19
1-butyl-3-methylimidazolium Dicyanamide	293.15	(Asensio-Delgado et al., 2021b)	1.92	2.46	6.59	5.09
1-butyl-3-methylimidazolium Dicyanamide	303.15	(Asensio-Delgado et al., 2021b)	2.4	3.02	9.12	6.08
1-butyl-3-methylimidazolium Dicyanamide	313.15	(Asensio-Delgado et al., 2021b)	3.1	3.66	12.29	7.15
1-butyl-3-methylimidazolium Dicyanamide	323.15	(Asensio-Delgado et al., 2021b)	3.94	4.38	15.96	8.30
1-butyl-3-methylimidazolium Thiocyanate	298.15	(Asensio-Delgado et al., 2021b)	3.11	2.89	13.32	6.95
1-hexyl-3-methylimidazolium Tetrafluoroborate	303.15	(He et al., 2017)	1.44	2.60	3.19	4.71
1-hexyl-3-methylimidazolium Bis(trifluoromethylsulfonyl)imide	298.15	(He et al., 2017)	0.96	2.15	1.46	3.01
1-ethyl-3-methylimidazolium Perfluoropentanoate	303.15	(Sosa et al., 2019)	1.17	2.29	0.93	2.80
1-ethyl-3-methylimidazolium Perfluorobutanesulfonate	303.15	(Sosa et al., 2019)	1.15	2.31	1.29	2.97
Trihexyltetradecylphosphonium Chloride	298.15	(Baca et al., 2021)	0.63	2.02	0.37	1.12

S25

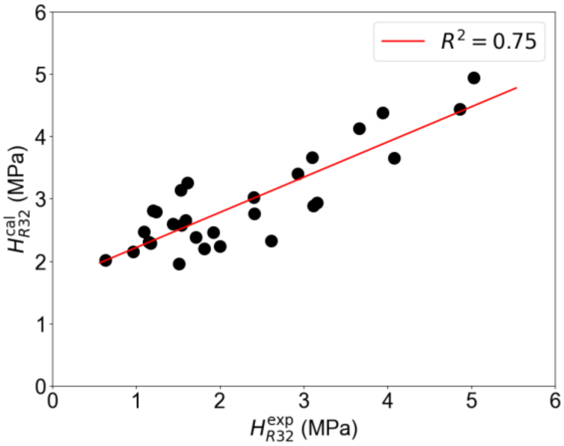

Figure S6: Experimental and COSMO calculated Henry's constants for R-32 in different ILs at different temperatures.

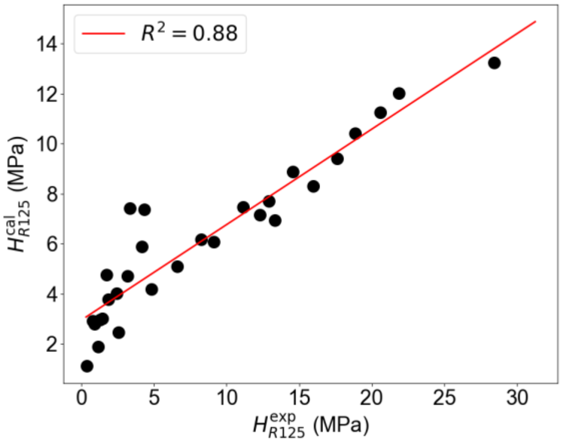

Figure S7: Experimental and COSMO calculated Henry's constants for R-125 in different ILs at different temperatures.

Reference

Asensio-Delgado, S., Pardo, F., Zarca, G., Urtiaga, A., 2021a. Absorption separation of fluorinated refrigerant gases with ionic liquids: Equilibrium, mass transport, and process design. Separation and Purification Technology 276, 119363. https://doi.org/10.1016/j.seppur.2021.119363

Asensio-Delgado, S., Viar, M., Pardo, F., Zarca, G., Urtiaga, A., 2021b. Gas solubility and diffusivity of hydrofluorocarbons and hydrofluoroolefins in cyanide-based ionic liquids for the separation of refrigerant mixtures. Fluid Phase Equilibria 549, 113210.

Baca, K.R., Olsen, G.M., Matamoros Valenciano, L., Bennett, M.G., Haggard, D.M., Befort, B.J., Garciadiego, A., Dowling, A.W., Maginn, E.J., Shiflett, M.B., 2021. Phase Equilibria and Diffusivities of HFC-32 and HFC-125 in Ionic Liquids for the Separation

of R-410A. ACS Sustainable Chemistry & Engineering 10, 816–830.
Demirel, S.E., Li, J., Hasan, M.M.F., 2017. Systematic process intensification using building blocks. Computers & Chemical Engineering 105, 2–38.
He, M., Peng, S., Liu, X., Pan, P., He, Y., 2017. Diffusion coefficients and Henry's constants of hydrofluorocarbons in [HMIM][Tf2N],[HMIM][TfO], and [HMIM][BF4]. The Journal of Chemical Thermodynamics 112, 43–51.
Iftakher, A., Aras, C.M., Monjur, M.S., Hasan, M.M.F., 2022. Data-driven approximation of thermodynamic phase equilibria. AIChE Journal 68, e17624.
Iftakher, A., Monjur, M.S., Leonard, T., Gani, R., Hasan, M.M.F., 2025. Multiscale high-throughput screening of ionic liquid solvents for mixed-refrigerant separation. Computers & Chemical Engineering 109138. https://doi.org/10.1016/j.compchemeng.2025.109138
Monjur, M.S., Iftakher, A., Hasan, M.M.F., 2022. Separation Process Synthesis for High-GWP Refrigerant Mixtures: Extractive Distillation using Ionic Liquids. Ind. Eng. Chem. Res. 61, 4390–4406. https://doi.org/10.1021/acs.iecr.2c00136
Morais, A.R.C., Harders, A.N., Baca, K.R., Olsen, G.M., Befort, B.J., Dowling, A.W., Maginn, E.J., Shiflett, M.B., 2020. Phase Equilibria, Diffusivities, and Equation of State Modeling of HFC-32 and HFC-125 in Imidazolium-Based Ionic Liquids for the Separation of R-410A. Industrial & Engineering Chemistry Research 59, 18222–18235. https://doi.org/10.1021/acs.iecr.0c02820
Sahinidis, N.V., 1996. BARON: A general purpose global optimization software package. J Glob Optim 8, 201–205. https://doi.org/10.1007/BF00138693
Shiflett, M.B., Harmer, M.A., Junk, C.P., Yokozeki, A., 2006. Solubility and diffusivity of difluoromethane in room-temperature ionic liquids. Journal of Chemical & Engineering Data 51, 483–495.
Shiflett, M.B., Yokozeki, A., 2008. Binary vapor–liquid and vapor–liquid–liquid equilibria of hydrofluorocarbons (HFC-125 and HFC-143a) and hydrofluoroethers (HFE-125 and HFE-143a) with ionic liquid [emim][Tf_2N]. Journal of Chemical & Engineering Data 53, 492–497.
Sosa, J.E., Ribeiro, R.P., Castro, P.J., Mota, J.P., Araújo, J.M., Pereiro, A.B., 2019. Absorption of fluorinated greenhouse gases using fluorinated ionic liquids. Industrial & Engineering Chemistry Research 58, 20769–20778.

S27
